# Occurrence and Prognosis of Mixed Subtype Adenocarcinoma and Adeno-Squamous Carcinoma in Esophageal Cancer

**DOI:** 10.7150/jca.92230

**Published:** 2024-01-20

**Authors:** Dengfeng Zhang, Tianxing Lu, Pengfei Guo, Jing li, Fangchao Zhao, Zhirong Li, Shujun Li

**Affiliations:** Department of Thoracic Surgery, The Second Hospital of Hebei Medical University, Shijiazhuang, China.

**Keywords:** adenocarcinoma with mixed subtypes, adeno-squamous carcinoma, esophagus cancer, Incidence, Prognostic, Nomogram.

## Abstract

**Purpose:** To gain a deeper understanding of the incidence and survival rates of rare esophageal mixed adenoacanthoma (EAM) and esophageal mixed adeno-squamous carcinoma (EASC) to promote a more comprehensive understanding of these two subtypes.

**Background:** EAM and EASC are rare subtypes of esophageal cancer with limited literature available. Extensive research has been conducted on the clinical and pathological characteristics of gastric and colorectal mixed adenoacanthomas, but there is relatively little literature on esophageal mixed adenoacanthomas. Therefore, this study aims to investigate the incidence and survival rates of these two subtypes in depth.

**Methods:** Patients diagnosed with EAM and EASC between 2000 and 2019 were selected from the SEER database for the study. Joinpoint software was used to calculate the incidence rates of esophageal AM and ASC patients, and differences in cancer overall survival (OS) and cancer-specific survival (CSS) based on Kaplan-Meier curves were compared. Multivariate Cox regression analysis was employed to identify independent prognostic factors for OS and CSS, and a prognostic model was established and validated for accuracy.

**Results:** The study found that the incidence of EAM increased until 2014, followed by a decline, while the incidence of EASC decreased until 2017, followed by an increase. Both of these subtypes were more common in male patients and those over the age of 65. For EAM patients, preoperative chemoradiotherapy was associated with better survival rates, while for EASC patients, preoperative radiotherapy combined with adjuvant chemotherapy improved survival. Finally, we constructed nomograms for predicting the overall survival of EAM and EASC patients by incorporating identified risk factors, which demonstrated good sensitivity and specificity.

**Conclusion:** EAM and EASC are rare subtypes of esophageal cancer, and an in-depth exploration of their incidence and survival rates provides valuable data and insights for understanding these rare esophageal cancer subtypes. This information can assist clinical decision-making for healthcare professionals.

## Introduction

According to Global cancer statistics, esophageal cancer (EC) ranks as the seventh most common cancer globally and the sixth leading cause of cancer-related deaths 1. Both esophageal squamous cell carcinoma (ESCC) and esophageal adenocarcinoma (EAC) predominantly affect males. While the incidence of esophageal squamous cell carcinoma has shown a noticeable decline in recent decades, esophageal adenocarcinoma is on the rise 2. The Annual Percentage Change (AAPC) for ESCC is -1.5 (95% CI -2.4, -0.7), whereas the AAPC for EAC is 5.2 (95% CI 4.2, 6.2) 3. The 5-year survival rate for EC remains one of the lowest among all cancer types, with a 20% 5-year survival rate reported in the United States 4. A major reason for the poor prognosis of esophageal malignancies is their tendency not to manifest symptoms until late stages of the disease 5. Adenocarcinoma subtypes dominate in countries such as the United States, Australia, the United Kingdom, and Western Europe (Finland, France, Norway) 6.

Mixed adenocarcinoma (AM) is an uncommon adenocarcinoma subtype characterized by the coexistence of conventional adeno-carcinomatous components with areas of differentiation 7,8, often featuring a combination of glandular and poorly cohesive or signet ring cells 9,10. While AM is a rare but highly aggressive histological subtype in colorectal cancer, it has been identified as an independent adverse prognostic marker for overall survival 11. In gastric cancer, AM exhibits greater invasiveness in early-stage gastric cancer compared to other histological types 12. However, reports on the incidence and prognostic data related to esophageal AM are limited.

Adeno-squamous carcinoma (ASC) is a rare tumor characterized by the presence of both adeno-carcinomatous (AC) and squamous cell carcinoma (SCC) components, with an aggressive nature 13. Hence, the World Health Organization's classification of digestive system tumors defines the pathological characteristics of esophageal adeno-squamous carcinoma as lesions in the esophagus containing significant SCC elements mixed with tubular AC elements 9,14. Esophageal adeno-squamous carcinoma accounts for approximately 3.1% of all adeno-squamous carcinomas in the body 15 and 1% of all esophageal cancers 16. Adenocarcinoma exhibits a tendency for local infiltration. Some patients may manifest tumor-related fever, and there is potential susceptibility to immune checkpoint inhibitor suppression 17,18. Although there have been some reports on the prognosis of esophageal adeno-squamous carcinoma 19, they are generally based on individual case reports or have limited sample sizes 20,21.

Because both EAM and EASC are rare pathological subtypes of EC, there is a paucity of relevant reports and data. However, due to increasing attention in recent years, this study primarily aims to obtain baseline data and survival information for EAM and EASC patients from the Surveillance, Epidemiology, and End Results (SEER) database. It compares the incidence rates and survival outcomes of EAM and EASC patients, followed by constructing predictive models for the survival of EAM and EASC patients based on risk factors identified through multivariate Cox regression analysis. Finally, the predictive models undergo feasibility assessment from various perspectives.

## Patients and Methods

### Study population

**Figure 1:**
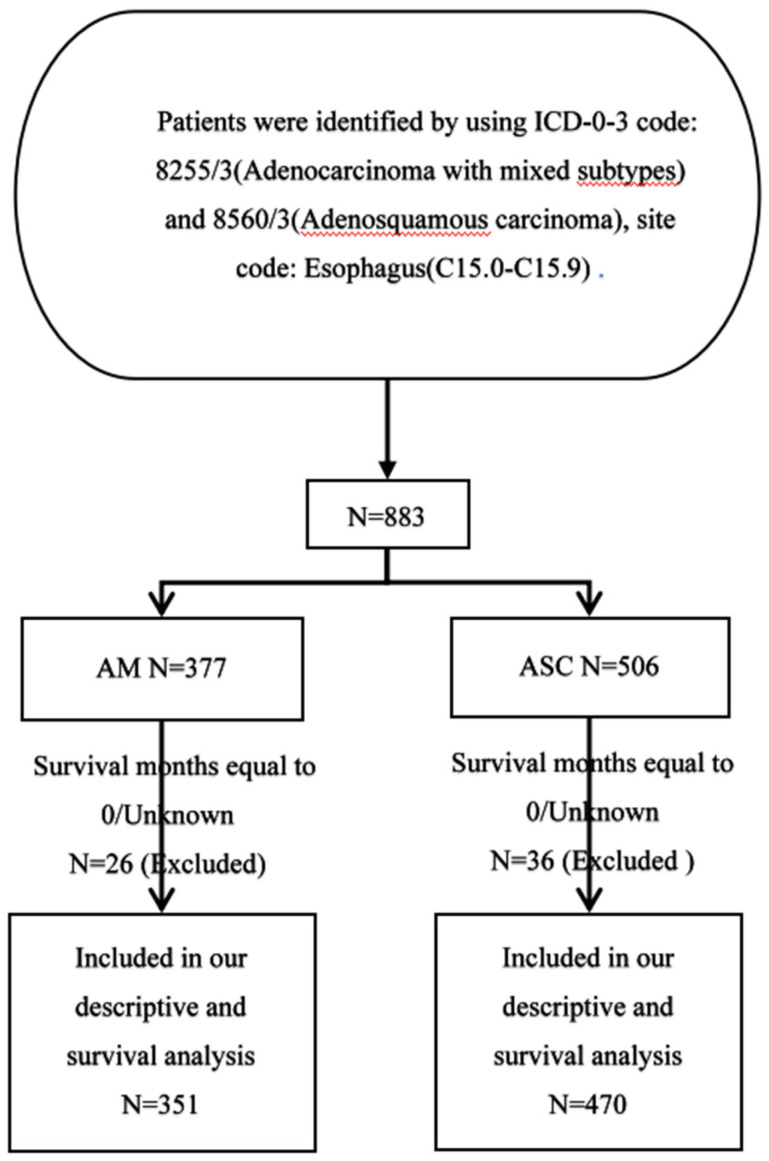
Flowchart of data screening.

In this study, all patient data were sourced from the Surveillance, Epidemiology, and End Results (SEER) database (https://seer.cancer.gov/), covering the period from 2000 to 2019. The SEER database collects and publishes data on cancer incidence and mortality from 18 cancer registries, encompassing approximately 28% of the population 22. The selection criteria for the study population were as follows: Primary site labeled (C15.1-15.9), pathological confirmation of Adenocarcinoma with mixed subtypes (ICD-O-3 8255/3), and Adenosquamous carcinoma (ICD-O-3 8560/3). At the same time, we excluded patients with missing or zero survival time data from the analysis **(Fig.1)**.

### Study Variables and Outcomes

In this study, the following variables were extracted from the SEER database: patient age, gender, race, tumor site, size, TNM staging (AJCC 7th edition), pathological grade, bone metastasis, liver metastasis, brain metastasis, lymph node metastasis, and metastasis to other sites (excluding bone, liver, brain, and lymph nodes), the number of lymph nodes removed, surgical status, chemotherapy status, and radiation therapy status. The primary endpoints of this study were overall survival (OS) and cancer-specific survival (CSS).

### Statistical Analysis

We calculated the incidence rates of esophageal adenocarcinoma (EAC) and esophageal squamous cell carcinoma (ESCC) patients between 2000 and 2019 using Joinpoint software (version 5.0.2), which can be accessed at https://surveillance.cancer.gov/joinpoint/. Subsequently, EAC and ESCC were stratified based on histology, and differences in cancer OS and CSS were compared using Kaplan-Meier survival curves. Multivariable Cox regression analysis was employed to identify independent prognostic factors for colorectal adenocarcinoma OS and CSS. All data analyses were conducted using R software version 4.2.3, available at https://www.r-project.org/.

## Results

### Patient Clinical and Tumor Characteristics

We selected a total of 377 patients diagnosed with esophageal adenocarcinoma mixed type and 506 patients diagnosed with esophageal squamous cell carcinoma between 2000 and 2009. There were significant differences between esophageal adenocarcinoma mixed type and esophageal squamous cell carcinoma in terms of race, diagnosis year, tumor location, pathological grade, AJCC Stage, TNM staging, and treatment modalities (surgery, lymph node dissection range), among others.

Compared to esophageal squamous cell carcinoma, patients with esophageal adenocarcinoma mixed type were more likely to be White (94.4% vs. 87.0%, P < 0.001), diagnosed in the years 2010-2019 (76.1% vs. 48.4%, P < 0.001), less likely to have tumors located in the middle third of the esophagus (7.2% vs. 21.1%, P < 0.001), more likely to be diagnosed at an early stage (I-II, 12.9% vs. 6.4%, P < 0.001), had larger tumor volumes (≥ 6cm, 12.2% vs. 8.7%, P < 0.136), a higher proportion of T3-T4 patients (T3-T4, 25.5% vs. 12.5%, P < 0.001), less frequent lymph node metastasis (N0, 15.6% vs. 8.3%, P < 0.001), and less frequent distant metastasis (M0, 34.7% vs. 16.8%, P < 0.001). More patients with esophageal adenocarcinoma mixed type underwent surgical treatment (Yes, 32.9% vs. 25.5%, P = 0.02), and a larger proportion had lymph node dissection involving >= 4 regional lymph nodes (28.4% vs. 15.8%, P < 0.001). Both types were more common in males. Detailed clinical and pathological characteristics can be found in **Table 1**.

### The incidence and trends of EAM and EASC

**Figure 2:**
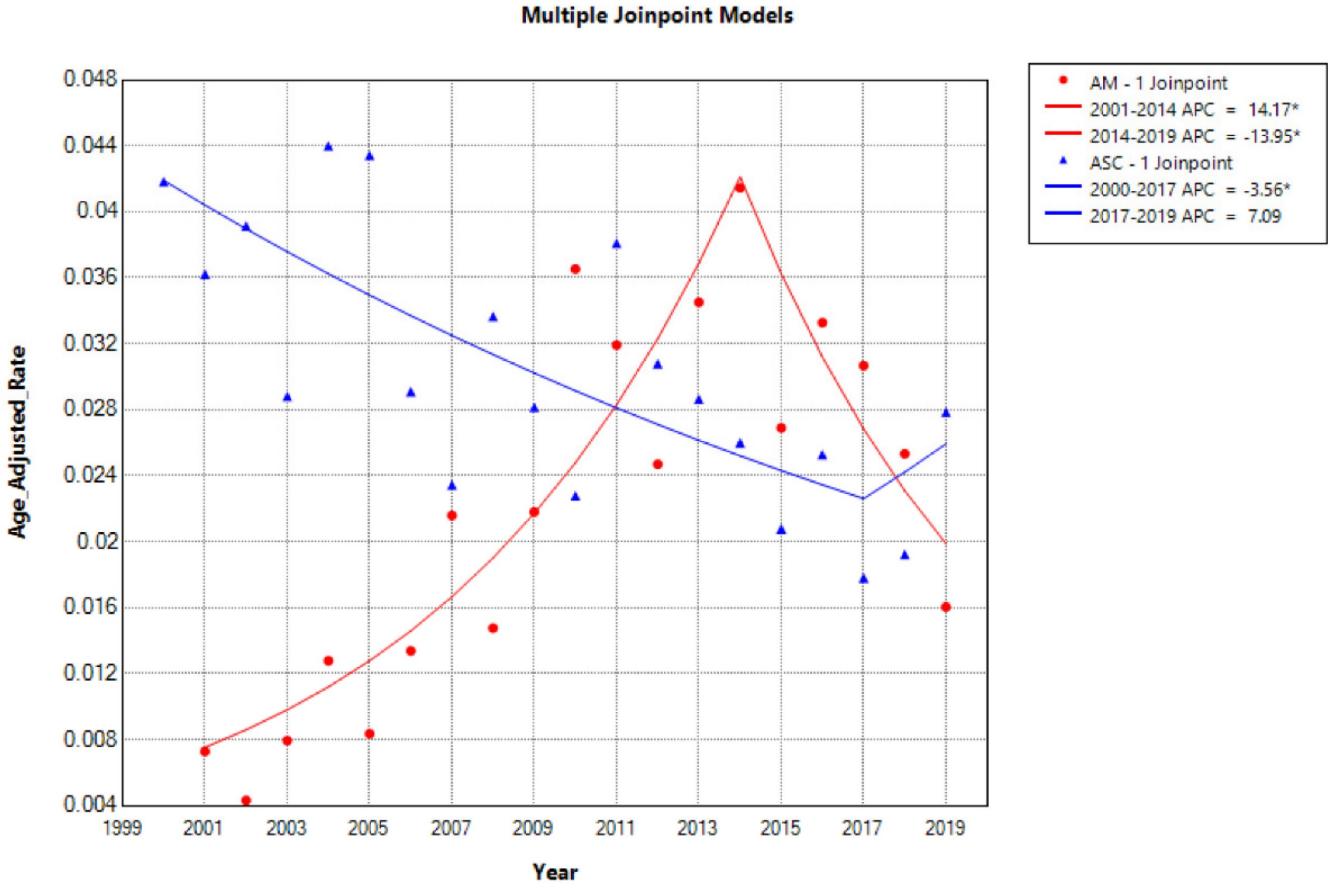
Trends in the Incidence of AM and ASC from 2000 to 2019 (* indicates p<0.05, Annual Percent Change (APC)).

The overall incidence and trends of esophageal adenocarcinoma (EAM) had been consistently increasing until 2014, followed by a subsequent decline. The highest overall incidence of EAM was 0.414 (95% CI: 0.292-0.571) per 100,000 person-years, while the lowest overall incidence was 0.043 (95% CI: 0.009-0.124) per 100,000 person-years. In contrast, the overall incidence and trends of esophageal squamous cell carcinoma (EASC) had been decreasing until 2017, after which they showed an increase. The highest overall incidence of EASC was 0.439 (95% CI: 0.302-0.618) per 100,000 person-years, while the lowest overall incidence was 0.178 (95% CI: 0.103-0.288) per 100,000 person-years **(Fig. 2)**.

### Esophageal Adenocarcinoma (EAM) and Esophageal Squamous Cell Carcinoma (EASC) - Comparison of Incidence Rates and Trends

#### Comparison of Incidence Rates in Different Subgroups of Esophageal Adenocarcinoma (EAM) Patients

In patients aged 65 and older, the incidence rate Average Annual Percent Change (AAPC) was 7.76% (2001-2019 AAPC = 7.76%, 95% CI 2.94%-16.39%, P < 0.05). In patients under 65 years old, the incidence rate was 7.58% (2001-2019 AAPC = 7.58%, 95% CI 3.20%-15.17%, P < 0.05). Incidence among males had an AAPC of 5.51% (2001-2019 AAPC = 5.51%, 95% CI 2.02%-10.28%, P < 0.05), while among females, it was 0.03% (2001-2019 AAPC = 0.03%, 95% CI -18.17%-22.27%, P = 0.99). Among ethnic groups, incidence rates for white individuals had an AAPC of 7.36% (2001-2019 AAPC = 7.36%, 95% CI 1.29%-18.03%, P < 0.05), black individuals had 2.19% (2001-2019 AAPC = 2.19%, 95% CI -5.89%-12.29%, P > 0.05), American Indian/Alaska Natives had 8.43% (2001-2019 AAPC=8.43%, 95% CI -10.96%-39.47%, P > 0.05), and Asian or Pacific Islanders had -0.73% (2001-2019 AAPC = -0.73%, 95% CI -5.30%-4.83%, P > 0.05). Regarding tumor location, incidence AAPC for upper 1/3 and middle 1/3 of the esophagus in EAM was -10.27% and -4.76%, respectively, whereas for the lower 1/3 of the esophagus, it was 6.40% (2001-2019 AAPC = 6.40%, 95% CI 1.11%-14.80%, P < 0.05).

#### Comparison of Incidence Rates in Different Subgroups of Esophageal Squamous Cell Carcinoma (EASC) Patients

**Figure 3:**
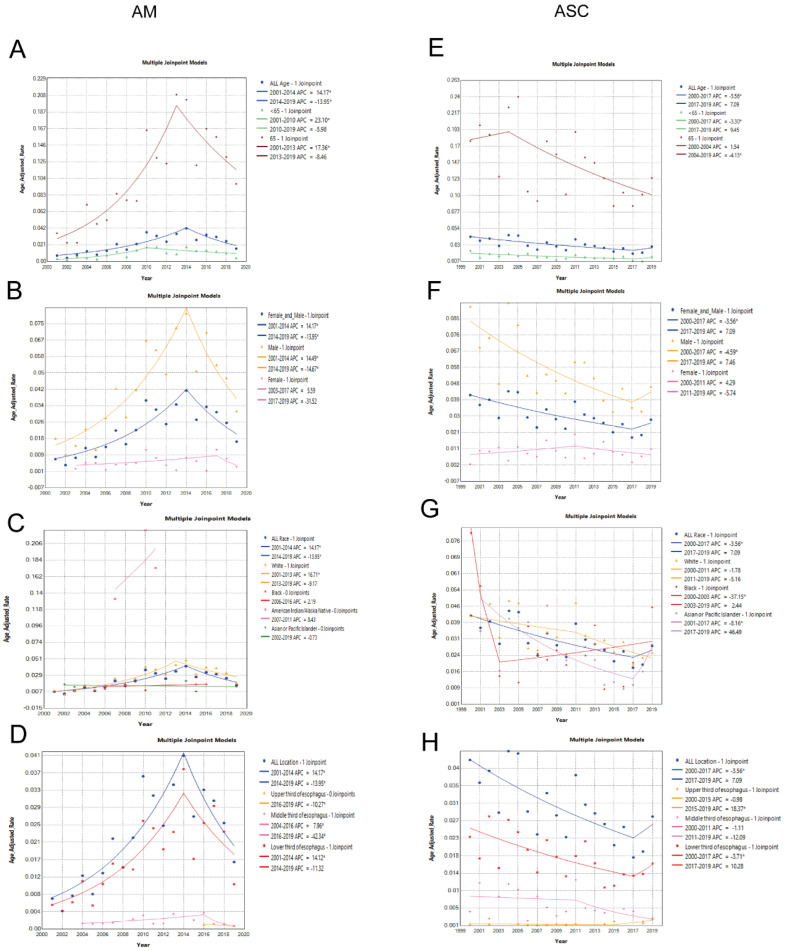
Differences in the incidence rates between AM and ASC subgroups. (A-D) Comparison of the incidence rates in subgroups based on age, sex, race, and tumor location among AM patients. (E-H) Comparison of the incidence rates in subgroups based on age, sex, race, and tumor location among ASC patients (* indicates p < 0.05).

In patients aged 65 and older, the incidence rate AAPC was -2.96% (2000-2019 AAPC = -2.96%, 95% CI -8.13%-2.50%, P = 0.28). In patients under 65 years old, the incidence rate was -2.03% (2000-2019 AAPC = -2.03%, 95% CI -7.57%-3.84%, P < 0.49). Incidence among males had an AAPC of -3.39% (2000-2019 AAPC = -3.39%, 95% CI -9.77%-3.44%, P = 0.32), while among females, it was -0.06% (2001-2019 AAPC = -0.06%, 95% CI -6.81%-7.19%, P = 0.99). Among ethnic groups, incidence rates for white individuals had an AAPC of -3.22% (2000-2019 AAPC = -3.22%, 95% CI -6.26%- -0.08%, P<0.05), black individuals had -5.17% (2000-2019 AAPC = -5.17%, 95% CI -8.73%- -0.68%, P < 0.05), and Asian or Pacific Islanders had -3.27% (2001-2019 AAPC = -0.73%, 95% CI -13.73%-8.47%, P = 0.57). Regarding tumor location, incidence AAPC for middle 1/3 and lower 1/3 of the esophagus in EASC were -5.89% and -2.32%, respectively, whereas for the upper 1/3 of the esophagus, it was 2.81% (2000-2019 AAPC = 2.81%, 95% CI 0.16%-5.27%, P < 0.05) **(Fig. 3A-H)**.

### Comparison of Survival between Different Histological Subtypes of Esophageal Cancer (EAM and EASC)

**Figure 4:**
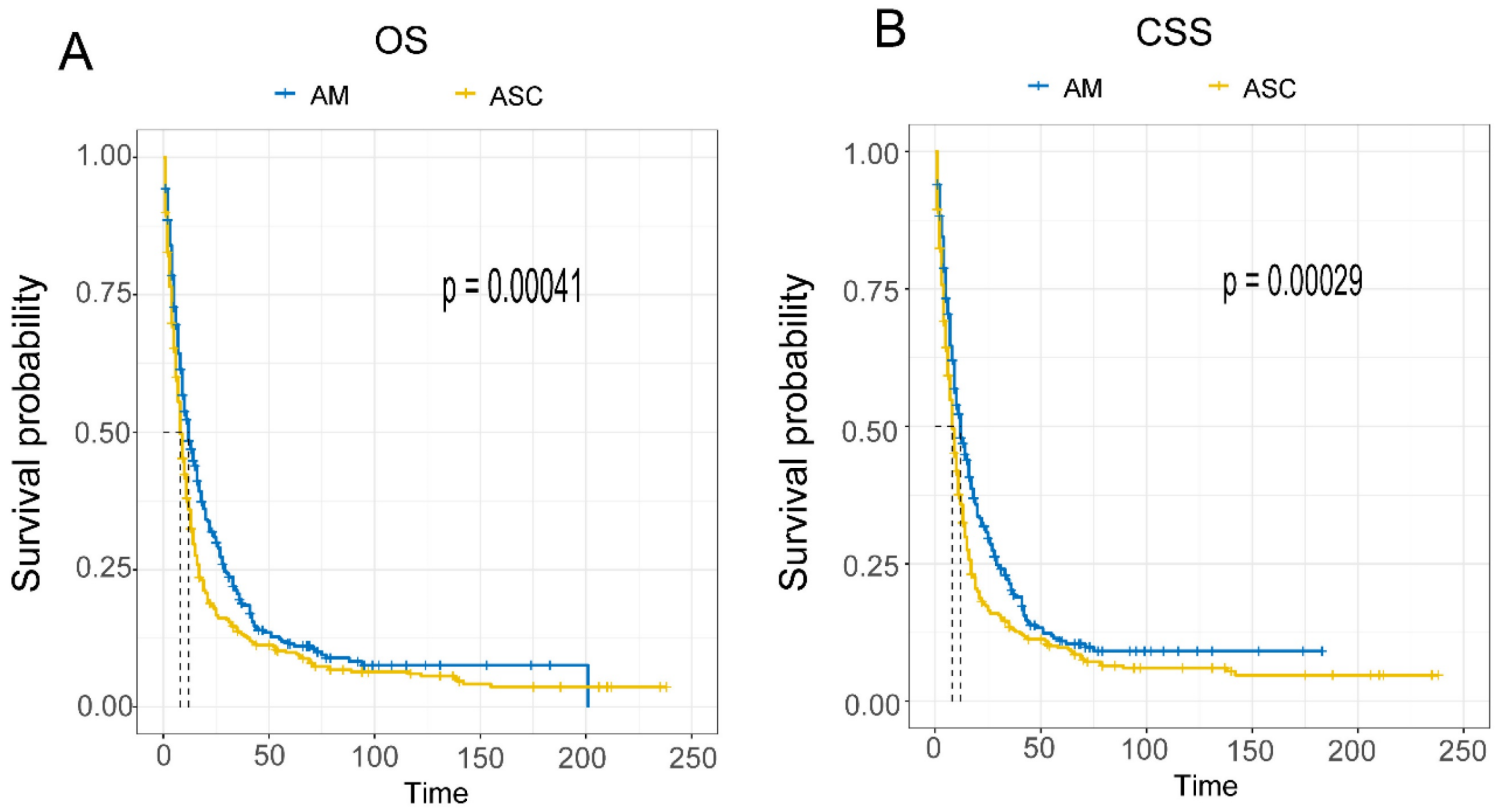
Comparison of Overall Survival (A) and Cancer-Specific Survival (B) between AM and ASC patients.

Using the Kaplan-Meier (K-M) method, the clinical outcomes of EAM were significantly superior to those of EASC, with a p-value < 0.001 **(Fig. 4A, B)**. Analytical results revealed that EAM patients had 1-year, 3-year, and 5-year overall survival rates (OS) of 48.4% (95% CI, 43.4%-54.0%), 20.2% (95% CI, 16.0%-25.5%), and 11.0% (95% CI, 7.7%-15.6%), respectively. The cancer-specific survival (CSS) rates for EAM patients at these time points were 47.9% (95% CI, 42.6%-53.8%), 19.6% (95% CI, 15.6%-24.5%), and 11.5% (95% CI, 8.4%-15.9%).For EASC patients, the OS rates were 35.9% (95% CI, 31.7%-40.6%), 13.5% (95% CI, 10.6%-17.2%), and 9.93% (95% CI, 7.43%-13.3%) at 1, 3, and 5 years, respectively, while the CSS rates were 35.8% (95% CI, 31.4%-40.8%), 13.2% (95% CI, 10.2%-17.1%), and 9.7% (95% CI, 7.4%-13.3%).

**Figure 5:**
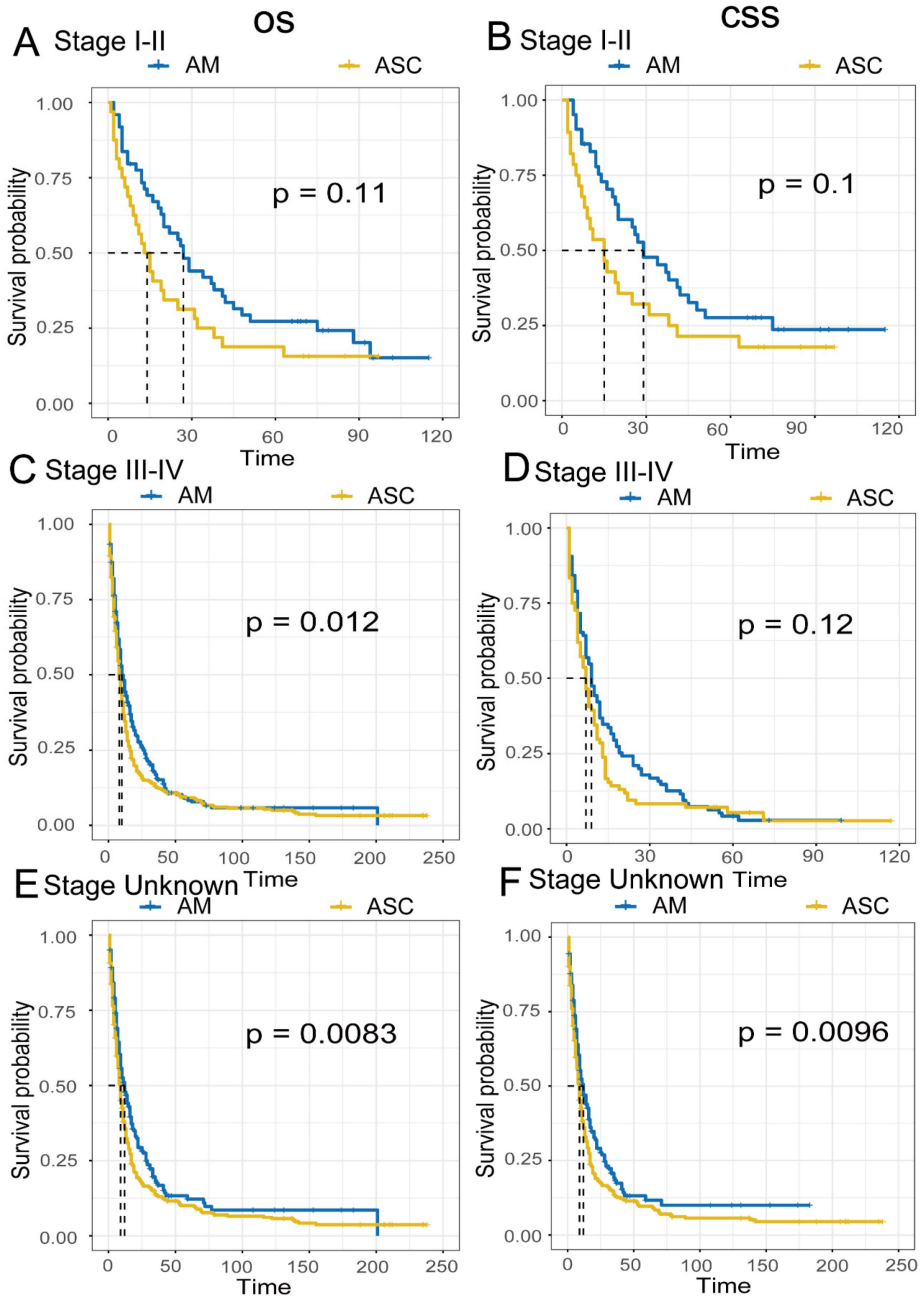
Comparison of Overall Survival (OS) and Cancer-Specific Survival (CSS) among AM and ASC patients in different AJCC stages.

Comparison of Survival Prognosis in Different AJCC Stages **(Fig. 5A-F)**. The results indicate that EAM patients in stages I-II had OS rates of 73.3% (95% CI, 61.9%-86.9%), 41.9% (95% CI, 30.0%-58.5%), and 27.2% (95% CI, 17.1%-43.3%) at 1, 3, and 5 years, respectively. The CSS rates for these patients were 77.8% (95% CI, 66.0%-91.7%), 45.2% (95% CI, 32.1%-63.6%), and 27.6% (95% CI, 16.7%-45.6%). In contrast, EAM patients in stages III-IV had OS rates of 44.3% (95% CI, 39.0%-50.4%), 15.6% (95% CI, 11.8%-20.7%), and 8.5% (95% CI, 5.5%-13.1%) at 1, 3, and 5 years, respectively. The CSS rates for these patients were 36.8% (95% CI, 28.3%-47.9%), 12.6% (95% CI, 7.4%-21.4%), and 4.2% (95% CI, 1.6%-11.0%). For EASC patients in stages I-II, the OS rates were 53.1% (95% CI, 38.4%-73.6%), 25.0% (95% CI, 13.7%-45.6%), and 18.8% (95% CI, 9.1%-38.6%) at 1, 3, and 5 years, respectively, while the CSS rates were 53.6% (95% CI, 37.9%-75.6%), 28.6% (95% CI, 15.9%-51.3%), and 21.4% (95% CI, 10.5%-43.6%). EASC patients in stages III-IV had OS rates of 34.6% (95% CI, 30.3%-39.5%), 12.6% (95% CI, 9.7%-16.4%), and 9.2% (95% CI, 6.7%-12.6%) at 1, 3, and 5 years, respectively. The CSS rates for these patients were 28.6% (95% CI, 20.4%-40.1%), 8.3% (95% CI, 4.1%-16.9%), and 5.4% (95% CI, 2.1%-13.9%).

**Figure 6:**
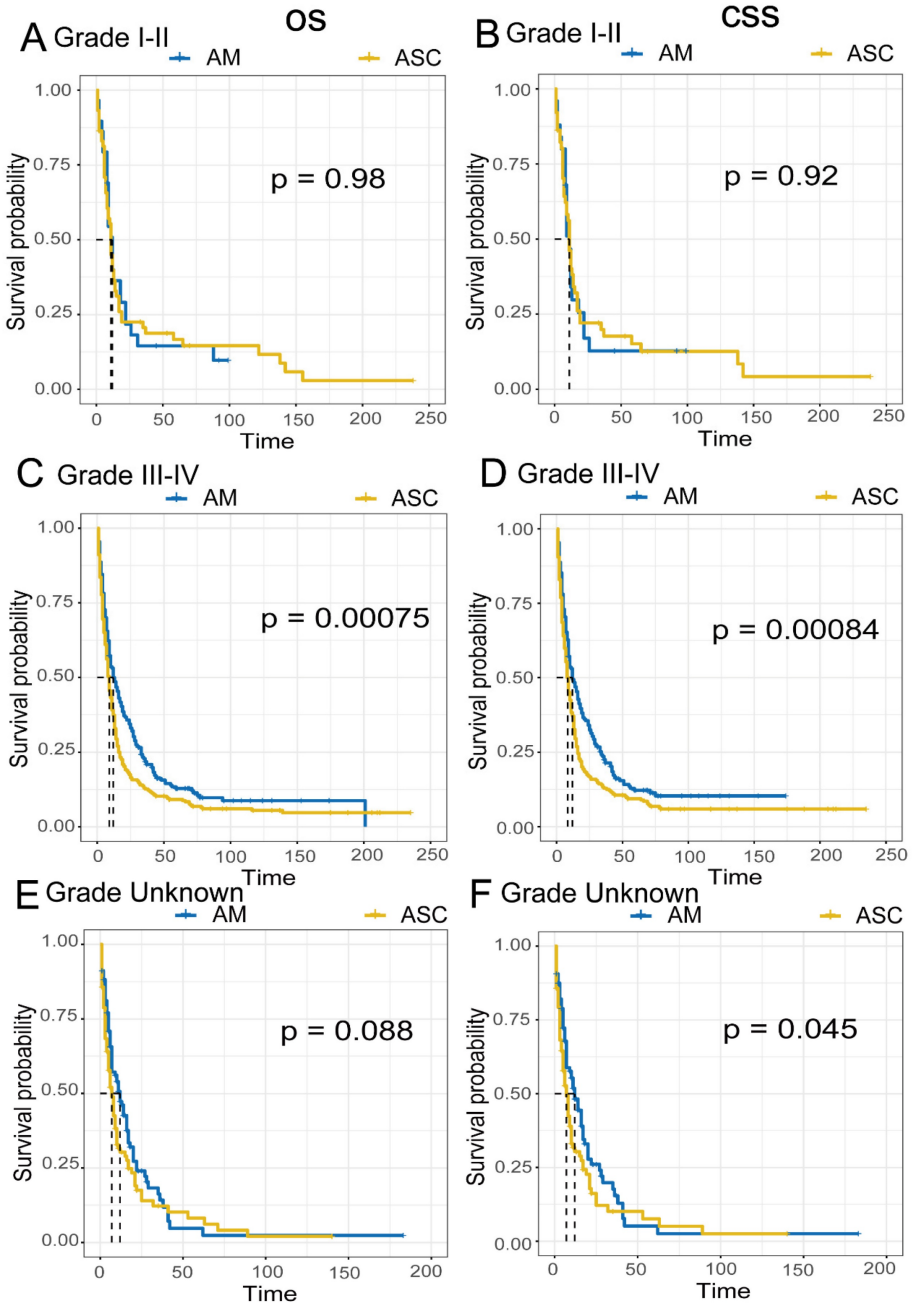
Comparison of Overall Survival (OS) and Cancer-Specific Survival (CSS) among AM and ASC patients in different pathological grades.

Comparison of Survival Prognosis in Different Pathological Grades **(Fig 6A-F)**. EAM patients in grades I-II had OS rates of 39.9% (95% CI, 25.3%-62.9%), 14.5% (95% CI, 5.9%-35.9%), and 14.5% (95% CI, 5.9%-35.9%) at 1, 3, and 5 years, respectively, with CSS rates of 34.0% (95% CI, 19.4%-59.5%), 12.7% (95% CI, 4.4%-36.6%), and 12.7% (95% CI, 4.44%-36.6%). For EAM patients in grades III-IV, the OS rates were 49.9% (95% CI, 43.8%-57.0%), 21.9% (95% CI, 17.0%-28.1%), and 12.9% (95% CI, 9.0%-18.4%) at 1, 3, and 5 years, respectively, with CSS rates of 49.4% (95% CI, 42.9%-56.9%), 22.5% (95% CI, 17.3%-29.2%), and 12.2% (95% CI, 8.2%-18.2%).

EASC patients in grades I-II had OS rates of 39.8% (95% CI, 29.0%-54.6%), 20.6% (95% CI, 12.4%-34.2%), and 16.6% (95% CI, 9.2%-30.0%) at 1, 3, and 5 years, respectively, with CSS rates of 40.1% (95% CI, 28.6%-56.3%), 19.9% (95% CI, 11.3%-34.8%), and 15.1% (95% CI, 7.7%-29.8%). EASC patients in grades III-IV had OS rates of 37.3% (95% CI, 32.2%-43.3%), 12.7% (95% CI, 9.4%-17.1%), and 9.2% (95% CI, 6.4%-13.2%) at 1, 3, and 5 years, respectively, with CSS rates of 37.1% (95% CI, 31.7%-43.4%), 12.9% (95% CI, 9.4%-17.6%), and 9.4% (95% CI, 6.4%-13.7%).

**Figure 7:**
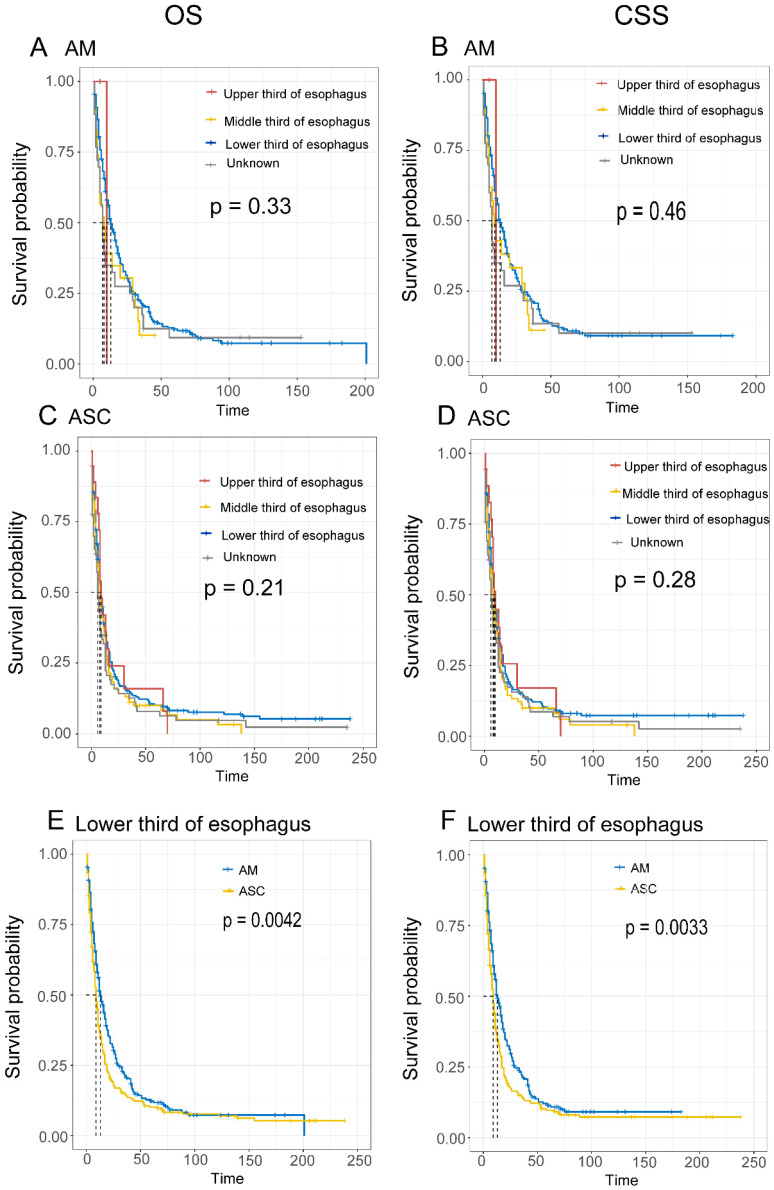
Comparison of Overall Survival (OS) and Cancer-Specific Survival (CSS) among AM and ASC patients in different tumor locations.

Comparison of Survival Based on Tumor Location. The results show that for EAM, when the tumor was located in the lower 1/3 of the esophagus, the best prognosis was observed, with OS rates of 51.4% (95% CI, 45.8%-57.6%), 20.7% (95% CI, 16.2%-26.3%), and 12.0% (95% CI, 8.5%-17.1%) at 1, 3, and 5 years, respectively, and CSS rates of 50.5% (95% CI, 44.7%-57.2%), 21.2%% (95% CI, 16.5%-27.3%), and 11.2% (95% CI, 7.5%-16.6%) **(Fig. 7A, B)**. For EASC, when the tumor was located in the upper 1/3 of the esophagus, the best prognosis was observed, with OS rates of 42.0% (95% CI, 24.0%-73.5%), 16.0% (95% CI, 5.0%-51.5%), and 16.0% (95% CI, 0.0498-0.515), and CSS rates of 0.448 (95% CI, 0.2597-0.772), 0.171 (95% CI, 5.0%-51.5%), and 17.1% (95% CI, 5.3%-54.5%) **(Fig. 7C, D)**. Additionally, when both EAM and EASC were located in the lower 1/3 of the esophagus, there were significant differences in terms of OS and CSS **(Fig. 7E, F)**.

**Figure 8:**
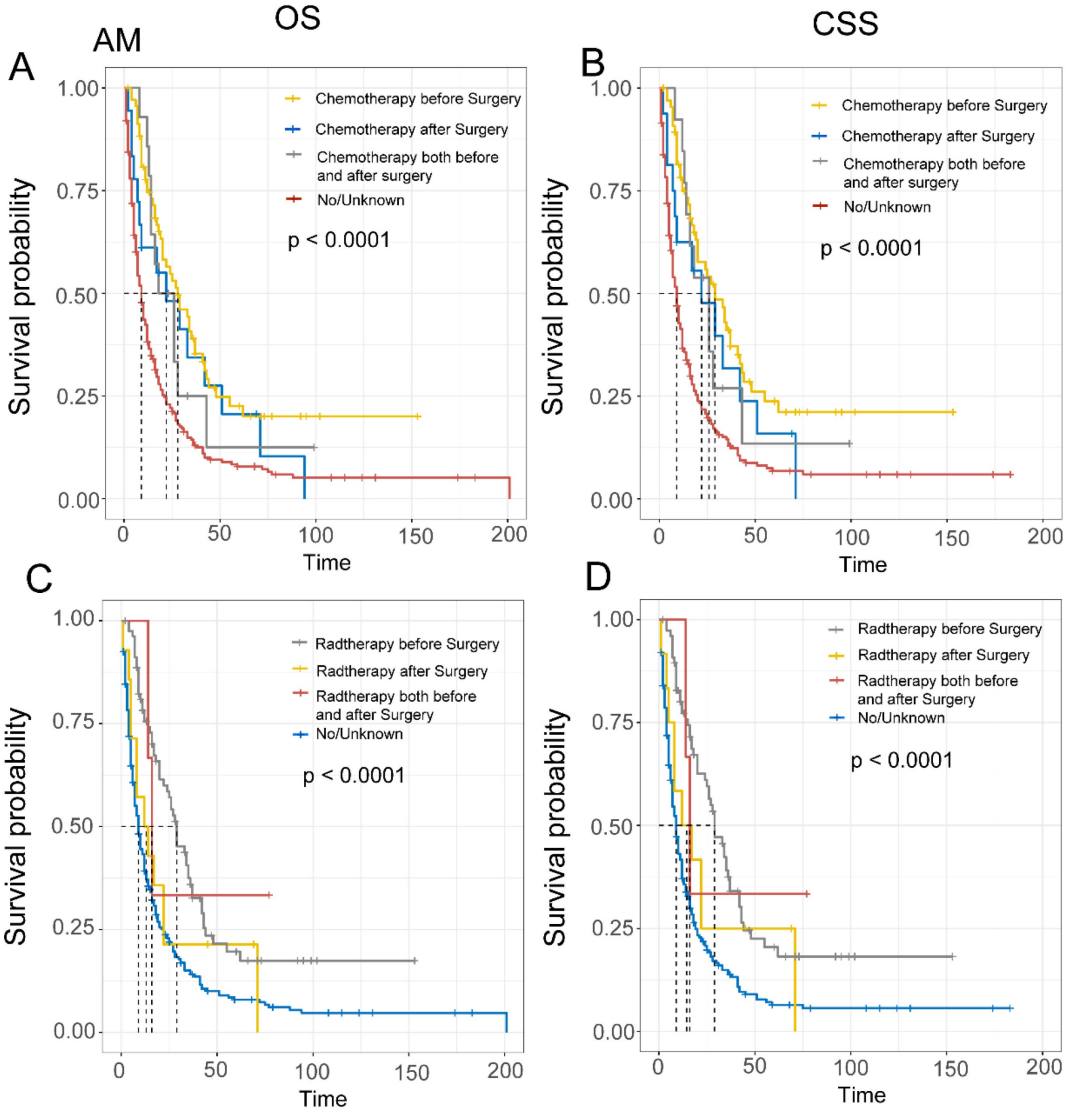
Comparison of Overall Survival (OS) and Cancer-Specific Survival (CSS) in AM patients with different sequences of radiotherapy and chemotherapy.

**Figure 9:**
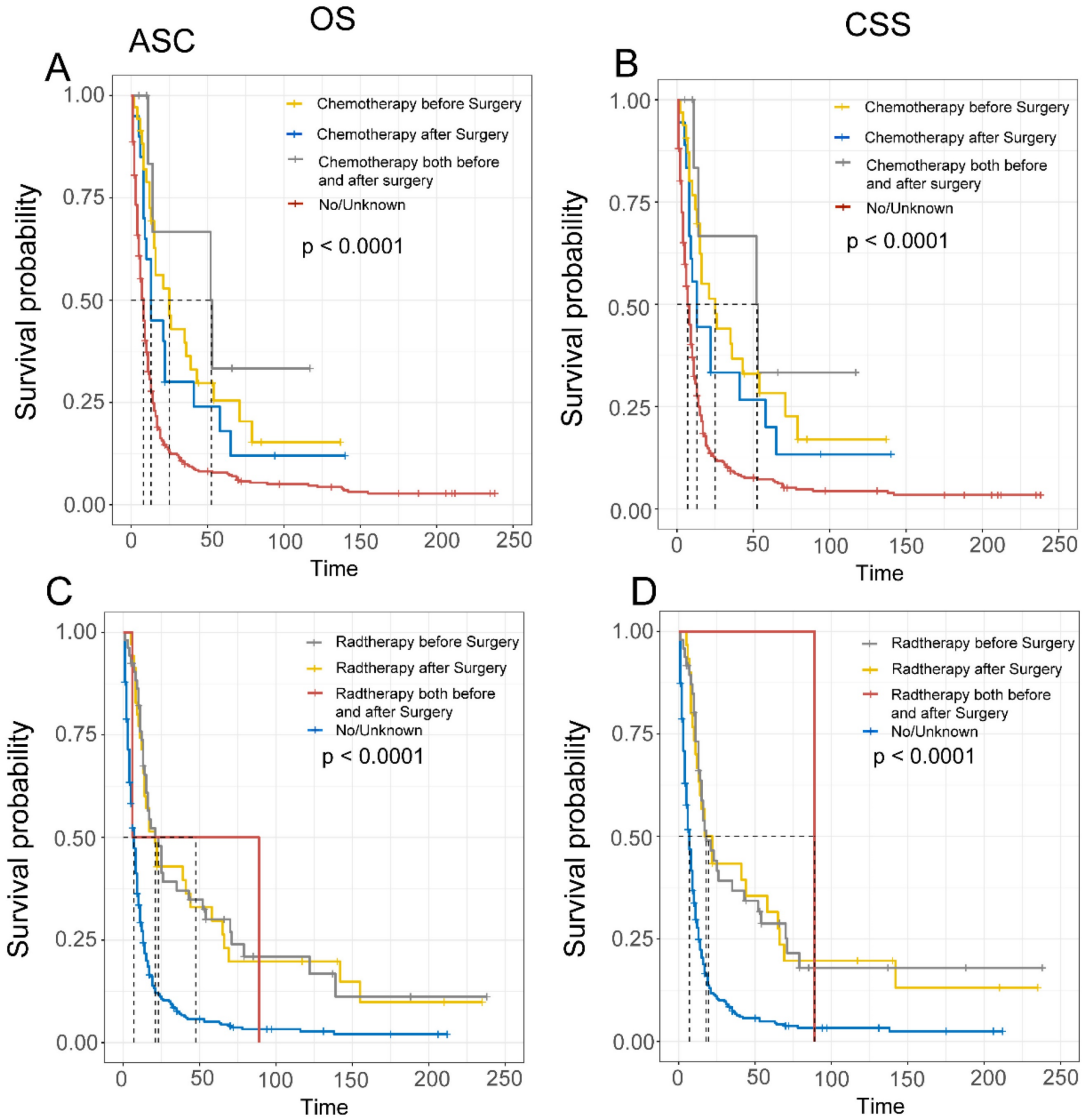
Comparison of Overall Survival (OS) and Cancer-Specific Survival (CSS) in ASC patients with different sequences of radiotherapy and chemotherapy.

Finally, we evaluated the impact of treatment sequencing on the prognosis of EAM and EASC patients. For EAM, patients who received preoperative radiation therapy had the highest benefits, with OS rates of 75.5% (95% CI, 66.5%-85.7%), 35.9% (95% CI, 26.1%-49.2%), and 19.6% (95% CI, 11.7%-32.8%) at 1, 3, and 5 years, respectively, and CSS rates of 77.2% (95% CI, 68.3%-87.4%), 37.4% (95% CI, 27.4%-51.1%), and 20.5% (95% CI, 12.3%-34.1%). Similarly, patients who received preoperative chemotherapy also had favorable outcomes, with OS rates of 74.6% (95% CI, 64.9%-85.8%), 39.0% (95% CI, 28.4%-53.5%), and 22.6% (95% CI, 13.7%-37.2%), and CSS rates of 75.0% (95% CI, 65.0%-86.4%), 41.1% (95% CI, 30.1%-56.0%), and 23.8% (95% CI, 14.4%-39.1%)** (Fig. 8A-D)**. In EASC, patients who received preoperative radiation therapy had the best outcomes, with OS rates of 73.8% (95% CI, 62.4%-87.1%), 37.0% (95% CI, 25.4%-53.8%), and 30.0% (95% CI, 19.2%-46.7%), and CSS rates of 73.1% (95% CI, 61.2%-87.4%), 36.7% (95% CI, 24.6%-54.7%), and 28.7% (95% CI, 17.7%-46.8%). Patients who underwent full-course chemotherapy also had favorable outcomes, with OS rates of 83.3% (95% CI, 58.3%-100.0%), 66.7% (95% CI, 37.9%-100.0%), and 33.3% (95% CI, 10.8%-100.0%), and CSS rates of 83.3% (95% CI,58.3%-100.0%), 66.7% (95% CI, 37.9%-100.0%), and 33.3% (95% CI, 10.8%-100.0%) **(Fig. 9A-D)**.

### The positivity rate of local lymph node involvement differs between subgroups of EAM and EASC patients

We calculated the local lymph node positivity rate for each patient by dividing the number of positive lymph nodes by the total number of lymph nodes examined. This rate was used for the subsequent analysis of various subgroups of EAM and EASC patients.

**Figure 10:**
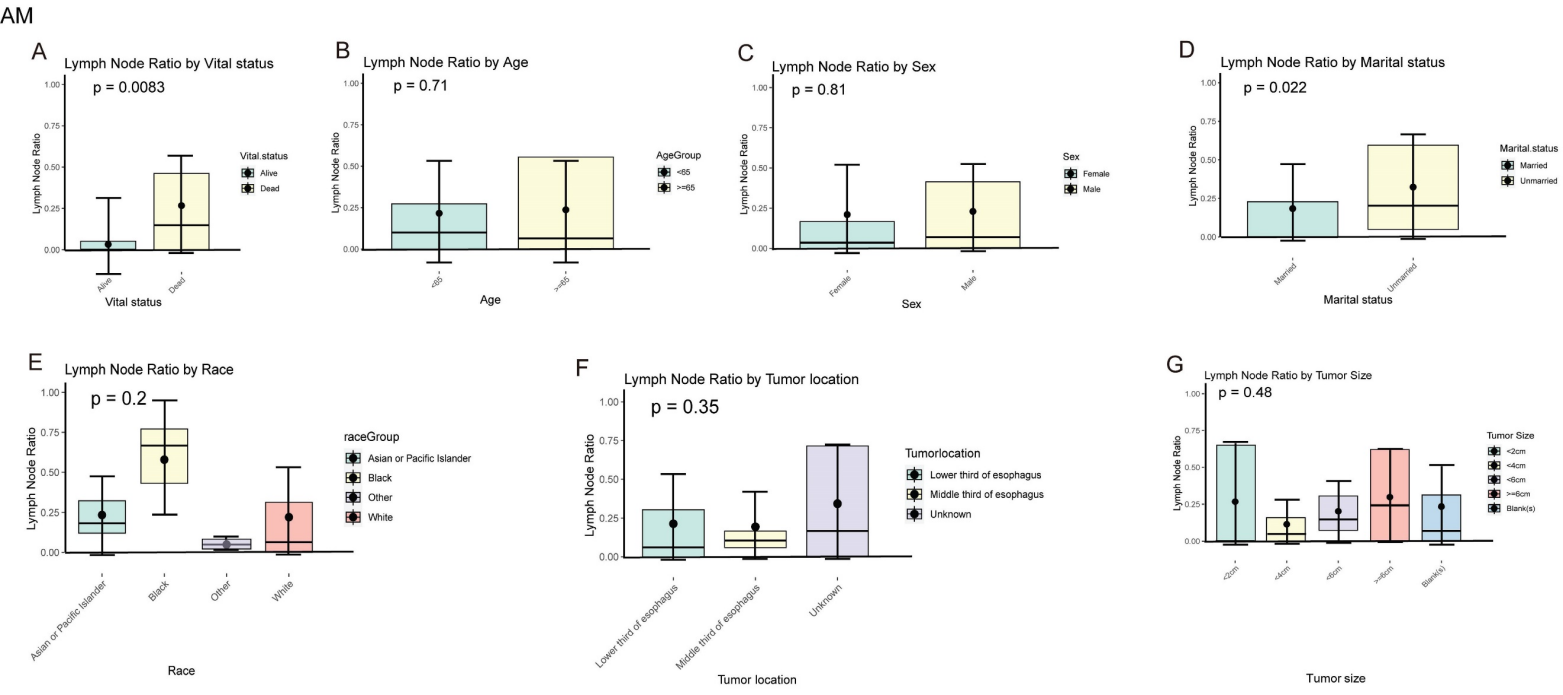
Positive local lymph node rates in different subgroups of AM.

In EAM patients, we compared the local lymph node positivity rates among different subgroups. The results showed that among the subgroups based on patient survival status, the local lymph node positivity rate was higher in the deceased group compared to the survival group (26.70% vs. 9.14%, P = 0.0083). Among patients aged 65 and older, the positivity rate was higher (≥ 65 vs. < 65, 23.68% vs. 21.61%, P = 0.710). The positivity rate was 22.97% in males compared to 21.05% in females (Male vs. Female, 22.97% vs. 21.05%, P = 0.810). Unmarried patients had a higher positivity rate compared to married patients (Unmarried vs. Married, 32.36% vs. 18.42%, P = 0.022). Black patients had the highest rate of local lymph node involvement, reaching 57.87%. When the tumor was located in the lower 1/3 of the esophagus, the positivity rate was highest at 21.34%. Tumors with a size of ≥ 6 cm had the highest local lymph node positivity rate, which was 29.78% **(Fig. 10A-G)**.

**Figure 11:**
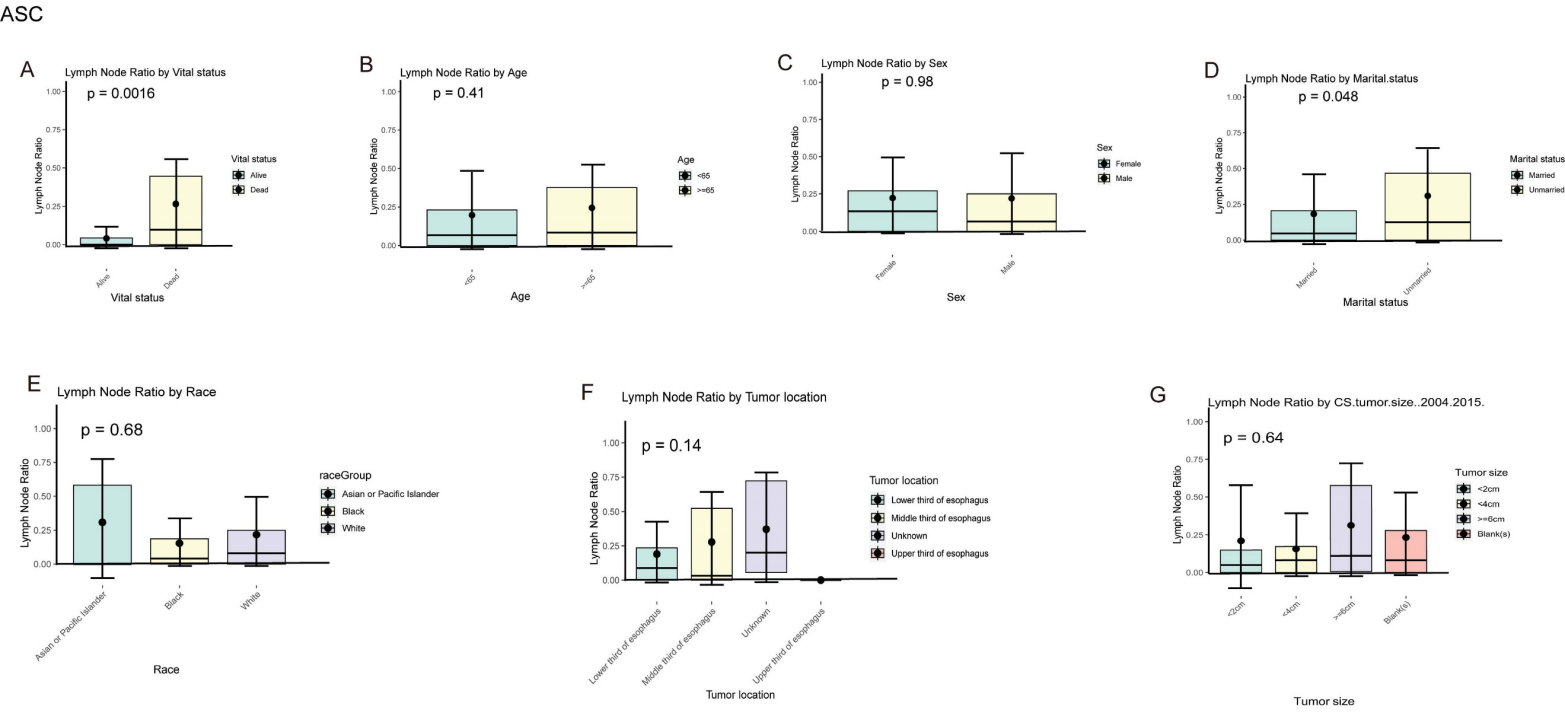
Positive local lymph node rates in different subgroups of ASC.

In EASC patients, the local lymph node positivity rate was 26.49% in the deceased group compared to 9.14% in the survival group (Dead vs. survival, 26.70% vs. 9.14%, P = 0.002). Among patients aged 65 and older, there was a higher likelihood of local lymph node involvement (≥ 65 vs. < 65, 24.48% vs. 19.73%, P = 0.410). The positivity rate was higher in females compared to males (Female vs. Male, 22.21% vs. 21.98%, P = 0.980). Asian or Pacific Islander patients had the highest rate of local lymph node involvement, reaching 30.90%. When the tumor was located in the middle 1/3 of the esophagus, the local lymph node positivity rate was highest at 27.79%. Tumors with a size of ≥ 6 cm were more likely to involve local lymph nodes, with a positivity rate of 31.21% **(Fig. 11A-G)**.

### Analysis of Risk Factors in EAM and EASC Patients

**Figure 12:**
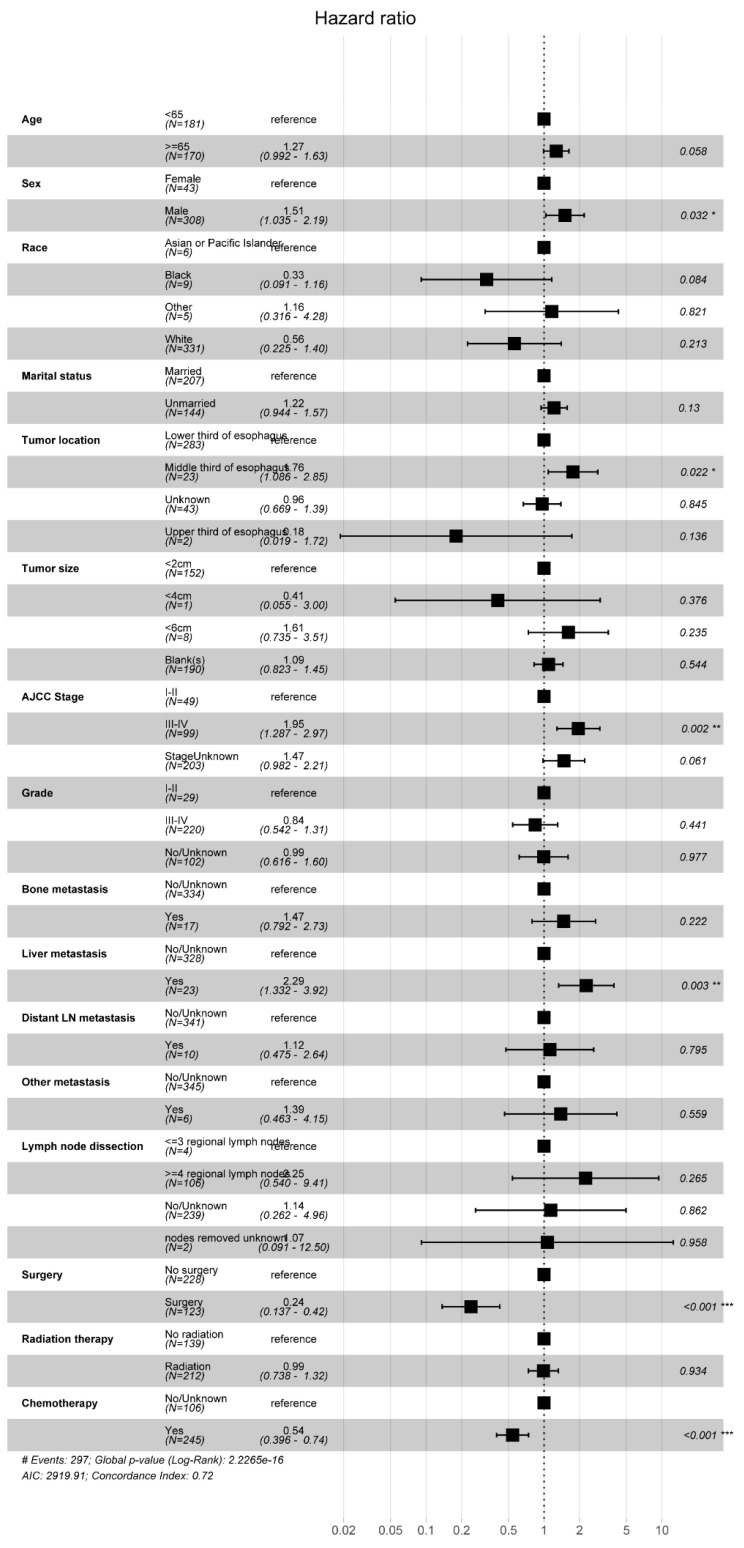
Forest Plot of Risk Factors Analysis in AM Patients.

We conducted a multifactorial Cox regression analysis to investigate risk factors in EAM patients. The results revealed a significant association between surgery, chemotherapy (Yes), and adverse overall survival (OS) outcomes (P < 0.001). Additionally, gender (P = 0.032, Male, HR = 1.51, 95% CI, 1.035-2.19), tumor location (P = 0.022, Middle third of esophagus, HR = 1.76, 95% CI, 1.086-2.85), AJCC Stage (P = 0.002, III-IV, HR = 1.95, 95% CI, 1.287-2.97), and liver metastasis (P = 0.003, Yes, HR = 2.29, 95% CI, 1.332-3.92) were also correlated with the prognosis of EAM patients. Notably, radiation therapy (P = 0.934, Yes, HR = 0.99, 95% CI, 0.738-1.32) emerged as a potential protective factor for EAM patients **(Fig. 12)**.

**Figure 13:**
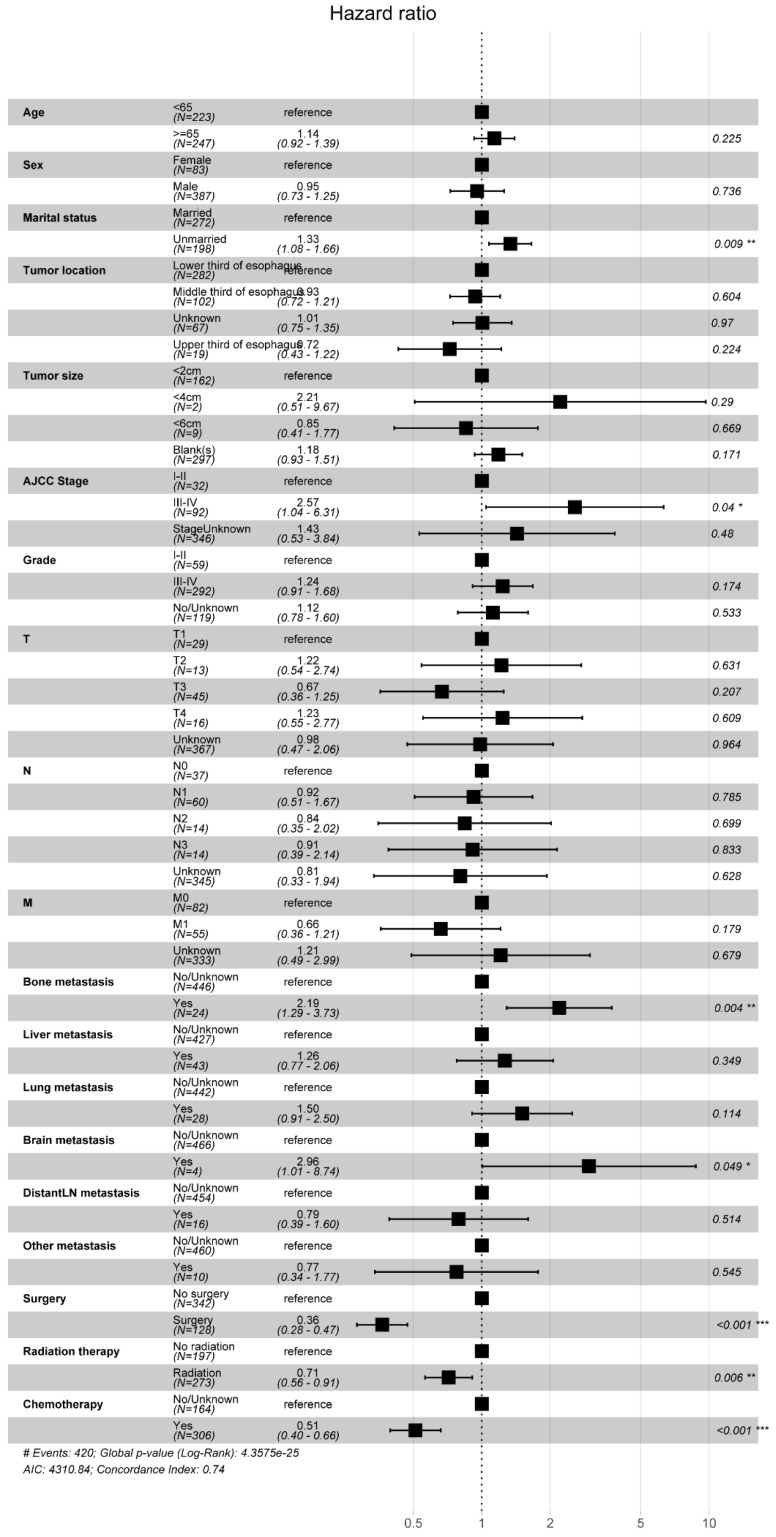
Forest Plot of Risk Factors Analysis in ASC Patients.

Similarly, we employed multifactorial Cox regression analysis to identify risk factors in EASC patients. The results indicated a significant association between surgery, chemotherapy (Yes), and adverse OS outcomes (P < 0.001).Marital status (P = 0.009, Unmarried, HR = 1.33, 95% CI, 1.08-1.66), AJCC Stage (P = 0.04, III-IV, HR = 2.57, 95% CI, 1.04-6.31), bone metastasis (P = 0.004, Yes, HR = 2.19, 95% CI, 1.29-3.73), brain metastasis (P = 0.049, Yes, HR = 2.96, 95% CI, 1.01-8.74), and radiation therapy (P = 0.006, Yes, HR = 0.71, 95% CI, 0.56-0.91) were identified as risk factors for EASC patients **(Fig. 13)**.

### Construction and Validation of OS Nomogram for EAM and EASC Patients

**Figure 14:**
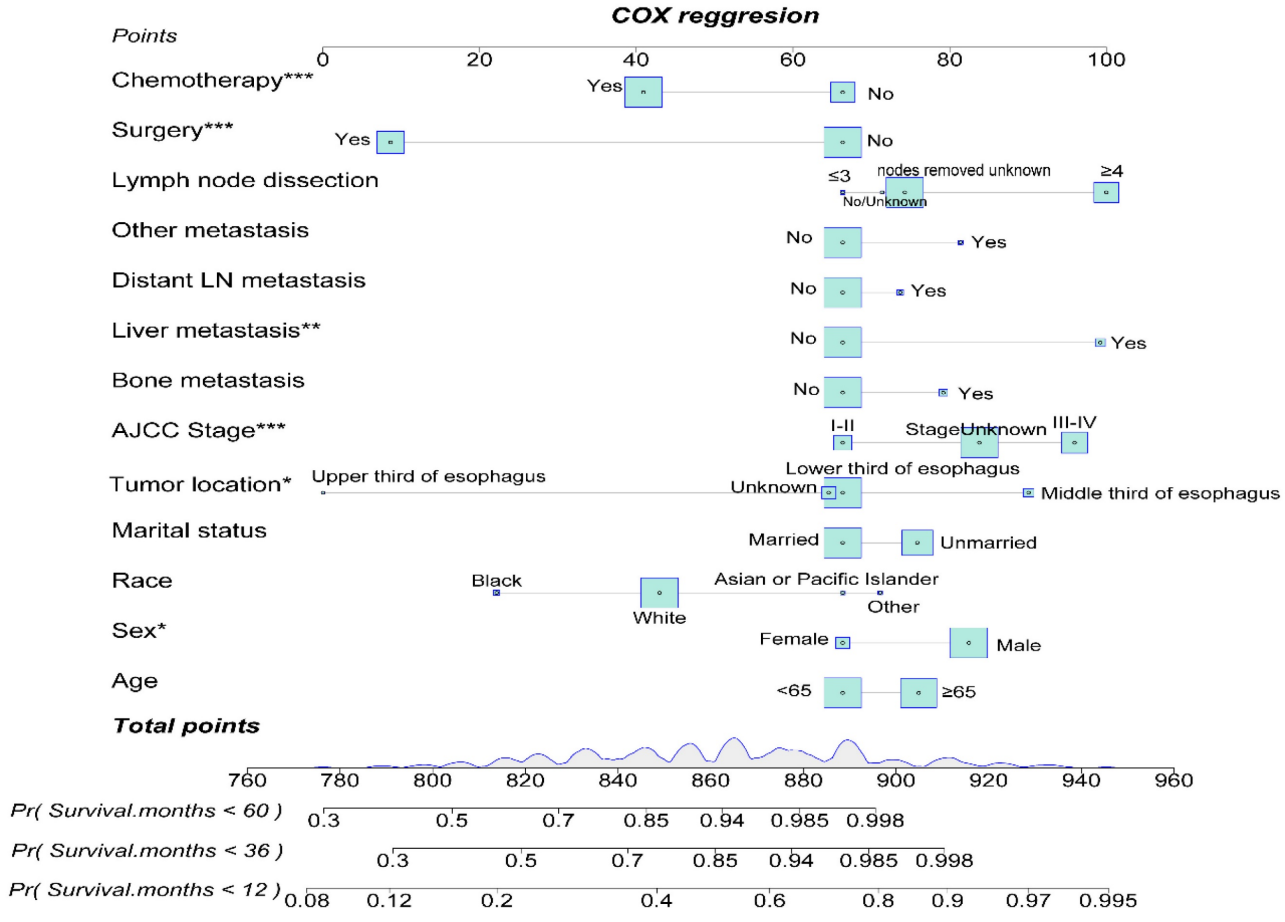
Construction of a Prognostic Model for AM Patients.

By utilizing multifactorial Cox regression analysis, we identified risk factors for EAM and included potential risk factors. Subsequently, we constructed a nomogram for predicting 1-year, 3-year, and 5-year overall survival (OS) for EAM patients based on variables including age, gender, race, marital status, tumor location, AJCC Stage, bone, liver, distant lymph node or other site metastasis, surgery, and chemotherapy **(Fig. 14)**. Each variable is assigned a corresponding score, and the total score obtained by summing all risk factors can be used to predict the 1-year, 3-year, and 5-year OS of EAM patients **(Supplementary Table 1)**.

**Figure 15:**
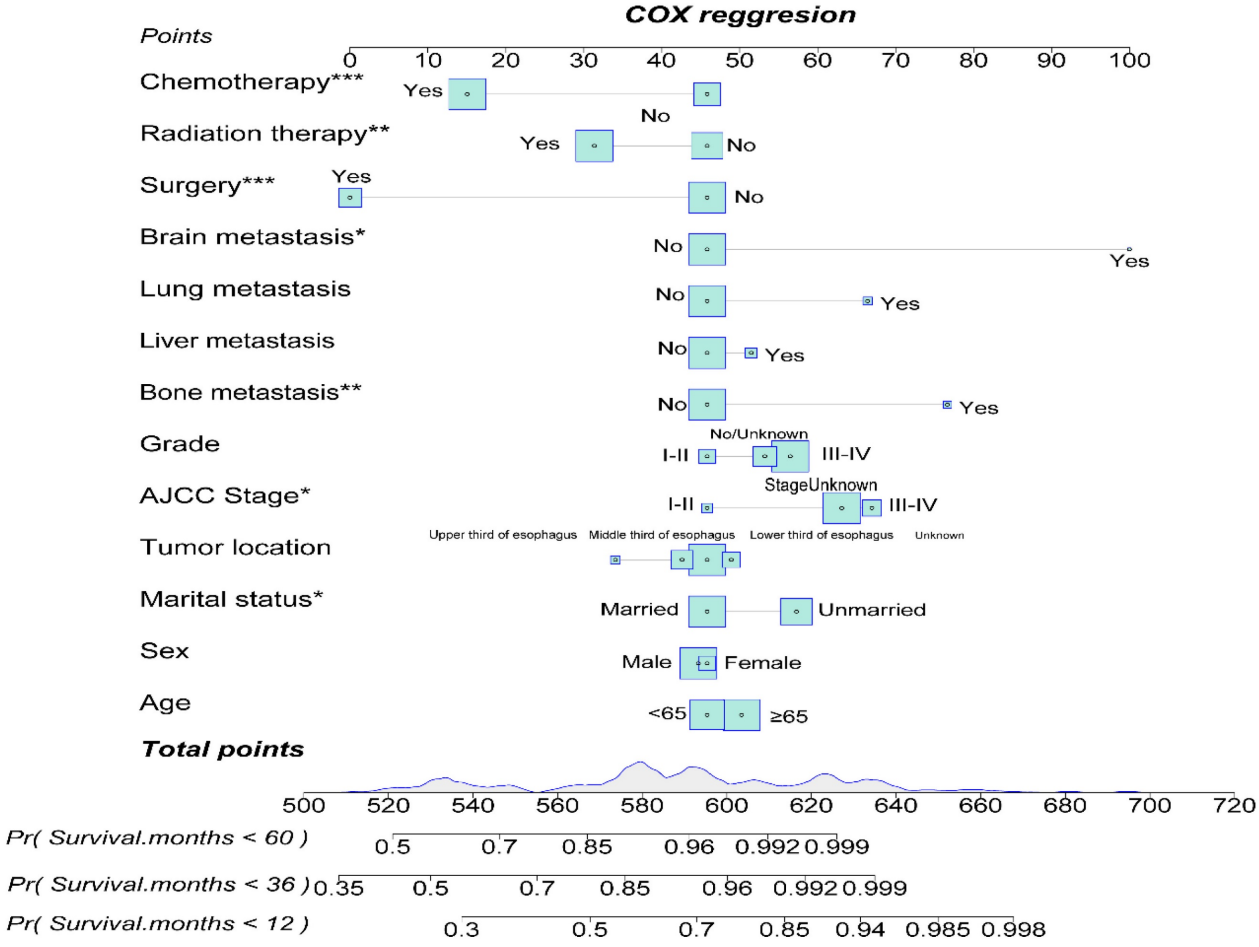
Construction of a Prognostic Model for ASC Patients.

The nomogram's corresponding C-index is 0.721, indicating good predictive ability. Similarly, in the EASC patient cohort, we included potential risk factors and constructed a nomogram for predicting 1-year, 3-year, and 5-year OS based on variables such as age, gender, marital status, tumor location, AJCC Stage, pathological grading, bone, liver, lung, brain metastasis, surgery, radiation therapy, and chemotherapy **(Fig. 15)**. Each variable has an associated score **(Supplementary Table 2)**. The nomogram's corresponding C-index is 0.731, indicating good predictive ability.

**Figure 16:**
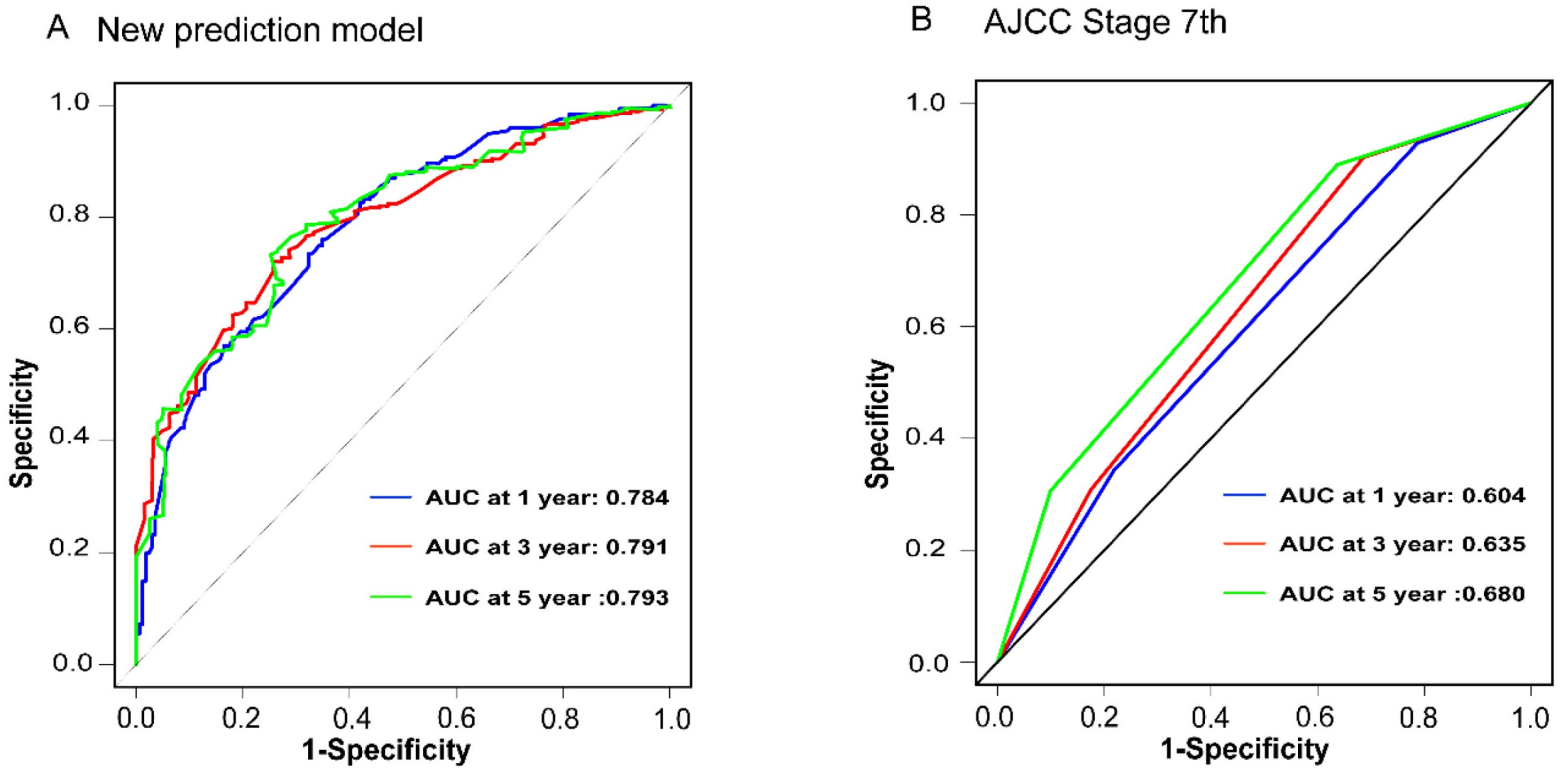
Comparison of ROC Curves between the New Predictive Model (A) for AM Patients and the AJCC Model (B).

**Figure 17:**
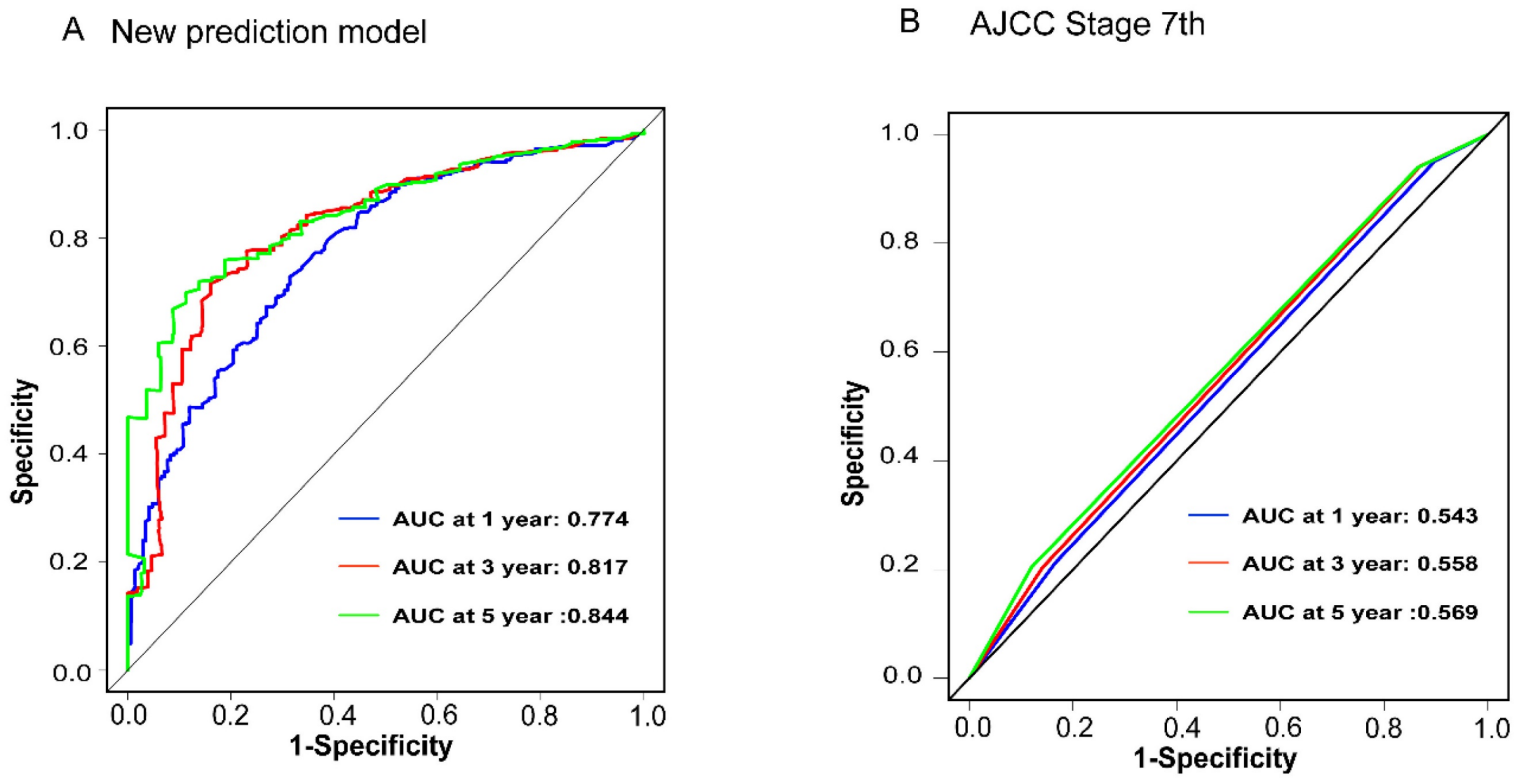
Comparison of ROC Curves between the New Predictive Model (A) for ASC Patients and the AJCC Model (B).

**Figure 18:**
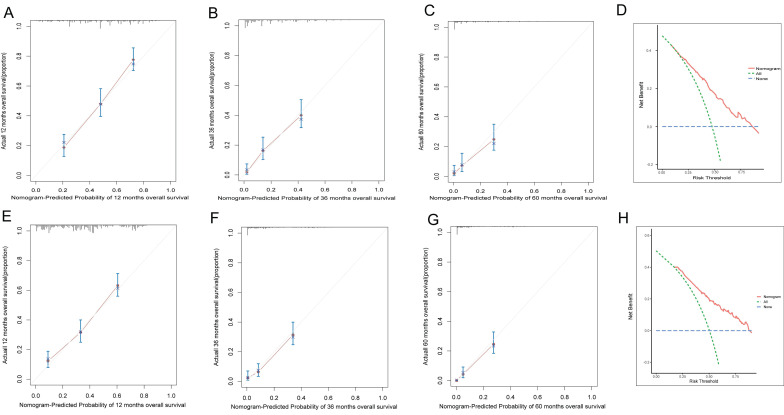
AM ASC patient prediction model calibration curves and DCA curves. (A-C) represent the calibration curves for the 12-month, 36-month, and 60-month AM prediction models. (D) represents the DCA curve for the AM prediction model, with the area under the curve indicating the net benefit. (E-G) represent the calibration curves for the 12-month, 36-month, and 60-month ASC prediction models. (H) represents the DCA curve for the ASC prediction model, with the area under the curve indicating the net benefit.

**Figure 19:**
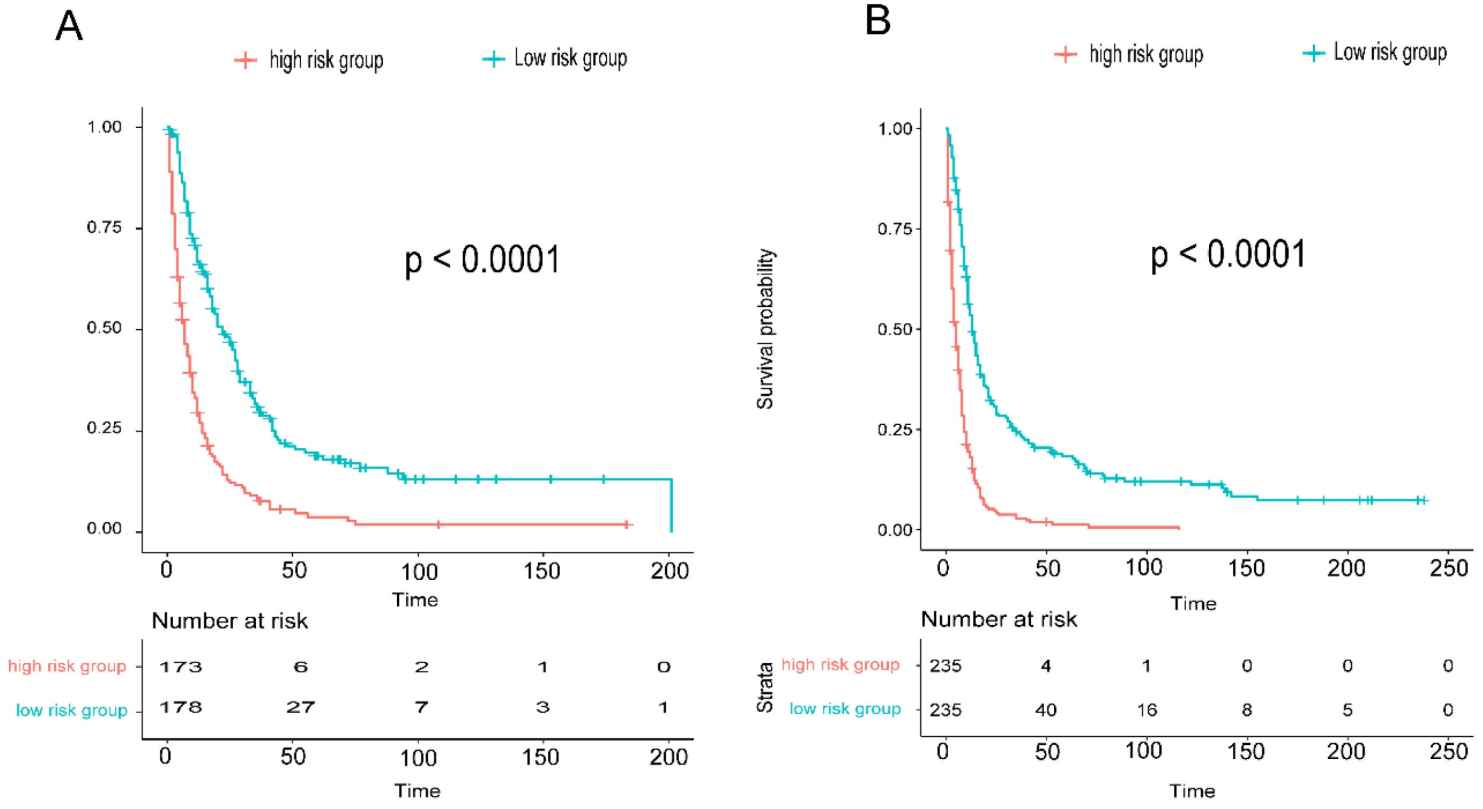
Comparison of Overall Survival (OS) between High and Low-Risk Groups Stratified by Predictive Models for AM (A) and ASC (B).

To assess the accuracy of the predictive models, ROC curves were plotted for 1-year, 3-year, and 5-year predictions. For EAM patients, the AUC values were 0.784, 0.791, and 0.793, respectively, which were superior to AJCC Stage 7 values of 0.604, 0.635, and 0.680 **(Fig. 16A, B)**. For EASC patients, the AUC values were 0.776, 0.818, and 0.844, respectively, also outperforming AJCC Stage 7 values of 0.543, 0.558, and 0.569 **(Fig. 17A, B)**. Subsequently, calibration curves were plotted to reveal high consistency between predicted and actual OS at 1 year, 3 years, and 5 years for both EAM **(Fig. 18 A-D)** and EASC **(Fig. 18 E-H)** patients. The decision curve analysis (DCA) results indicated a good net benefit. Finally, the predictive models were used to score EAM and EASC patient cohorts, categorizing patients into high and low-risk groups, demonstrating good predictive and discriminatory ability **(Fig. 19)**.

## Discussion

The reported incidence of EAM and EASC is limited, with a primary focus on the incidence of colorectal AM 8 or pancreatic ASC 23. Both EAM and EASC are rare subtypes of esophageal cancer, with extensive research conducted to report the clinical and pathological characteristics of gastric and colorectal AM. However, there is limited literature on esophageal AM 8,24. The prognosis of both types is currently unclear, and this study aims to promote a better understanding of the incidence and survival rates of esophageal AM and EASC.

The overall incidence and trends of esophageal AM increased until 2014 and then declined, with the highest overall incidence reaching up to 0.414 per 100,000 person-years. The overall incidence and trends of EASC decreased until 2017 and then increased, with the highest overall incidence reaching up to 0.439 per 100,000 person-years, which is consistent with the reported incidence of ASC ranging from 0.37% to 1% 25. In the overall incidence, EASC is more common than EAM, and there has been an upward trend in recent years (2017-2019 APC = 21). In both subtypes of esophageal cancer, patients aged 65 and older and male patients had a much higher incidence rate than the overall population, providing more targeted recommendations for our prevention measures. Furthermore, EASC patients had lower 1, 3, and 5-year overall survival (OS) and cancer-specific survival (CSS) compared to EAM, suggesting that EASC has a higher malignancy than EAM. Another possible reason is that EAM patients are mostly younger than 65 years old, and the tumor size is mostly ≤ 4 cm (EAM VS. EASC, Tumor size, 19.1% VS 16.4%).

In the comparison of survival prognosis at different AJCC stages, EAM patients in stages I, II, III, and IV had better OS and CSS than EASC patients, but significant differences were only observed in the OS of stages III-IV. In the comparison of survival prognosis at different pathological grades, EAM patients in grades I, II, III, and IV had better OS and CSS than EASC patients, with significant differences in the OS and CSS of grades III-IV. The composition of AM includes SRCC 26,27, and SRCC patients are mostly diagnosed at an advanced stage 28,29. According to our study, EAM patients were mostly in Grade III-IV (III-IV VS. I-II, 62.0% VS 7.9%) and Stage III-IV (III-IV VS. I-II, 29.2% VS 13.9%) at diagnosis, indicating that EAM also has similar characteristics to SRCC. In EASC, most patients were also in Grade III-IV (III-IV VS. I-II, 61.9% VS 12.1%) and Stage III-IV (III-IV VS. I-II, 19.9% VS 6.4%) at diagnosis, indicating that most patients were also in an advanced stage. Compared to EAM, more EASC patients were diagnosed at an early stage (EAM VS. EASC, Stage I-II, 12.9% VS 6.4%), and a larger number of regional lymph nodes were cleared (EAM VS. EASC, ≥4 regional lymph nodes, 28.4% VS 15.8%), further suggesting that the prognosis of EASC patients is worse than that of EAM patients. Cancer screening, early detection, early diagnosis, and early treatment are considered the most effective methods for cancer control and prevention in the world 30, and early cancer screening and diagnosis are especially important for patients who are already in an advanced stage at diagnosis, whether it is EAM or EASC.

In the survival prognosis comparison of tumor locations in EAM and EASC, we divided the esophagus into upper 1/3, middle 1/3, and lower 1/3. The results showed that EAM had a better prognosis when located in the lower 1/3 of the esophagus, while the prognosis was worst when located in the middle 1/3 of the esophagus. When EAM was located in the lower 1/3 of the esophagus, the local lymph node positivity rate was 21.34%, indicating that EAM in the lower 1/3 of the esophagus is more likely to have local lymph node metastasis. EASC had the best prognosis when located in the upper 1/3 of the esophagus and the worst prognosis when located in the middle 1/3 of the esophagus. When EASC was located in the upper 1/3 of the esophagus, there was no local lymph node positivity, while when located in the middle 1/3 of the esophagus, the local lymph node positivity rate reached as high as 27.70%, indicating that the middle 1/3 of the esophagus in EASC is more likely to have local lymph node metastasis, and prognosis is closely related to the local lymph node positivity rate.

The main location of ASC is still controversial in the literature, with some suggesting a preference for squamous cell carcinoma characteristics in the middle part of the esophagus 31,32, while others suggest a preference for adenocarcinoma characteristics in the distal esophagus 13,25. In our data, 60.3% of patients were located in the lower 1/3 of the esophagus, indicating a preference for adenocarcinoma characteristics.

Both EAM and EASC patients are prone to liver metastasis, with EAM patients also having bone (5.8%), lung (3.7%), and distant lymph node metastasis (2.9%), while EASC patients have lung (6.9%), bone (5.3%), and distant lymph node metastasis (3.2%). In comparing the local lymph node positivity rates in different subgroups, EAM is more likely to have local lymph node metastasis in patients aged ≥ 65 years, males, Black individuals, and those with tumor size ≥ 6 cm. On the other hand, EASC has a higher local lymph node positivity rate in patients aged ≥ 65 years, females, Asian or Pacific Islanders, and those with tumor size ≥ 6 cm.

As mixed subtype adenocarcinoma (AM) is a rare type of adenocarcinoma with a combination of glandular and poorly cohesive cell components, there is limited literature on esophageal mixed subtype adenocarcinoma (EAM). In our study, EAM was more common in younger individuals, males, White individuals, those with larger tumor volumes (≥6 cm), and mostly located in the lower 1/3 of the esophagus. Factors such as sex, tumor location, AJCC Stage, liver metastasis, surgery, chemotherapy, and radiation therapy were associated with the prognosis of EAM patients. In terms of treatment strategies, we explored the impact of different treatment regimens on tumor prognosis. The results showed that preoperative chemotherapy and radiation therapy provided the greatest benefit to EAM patients, with OS rates of 75.5%, 35.9%, and 19.6% at 1, 3, and 5 years for preoperative radiation therapy patients and OS rates of 74.6%, 39.0%, and 22.6% for preoperative chemotherapy patients. Subsequently, by constructing line charts, we predicted the 1, 3, and 5-year OS of EAM patients to assist clinical physicians in making better treatment decisions.

Esophageal adeno-squamous carcinoma (EASC) is also a mixed-type cancer characterized by a combination of adenocarcinoma (AC) and squamous cell carcinoma components 33. Our study shows that EASC is more common in elderly individuals (≥ 65 years old), males, White individuals, those with smaller tumor sizes (< 6 cm), and mostly located in the lower 1/3 of the esophagus. Marital status, AJCC Stage, bone metastasis, brain metastasis, surgery, chemotherapy, and radiation therapy are risk factors for EASC patients. The CSS of ASC patients depends on the primary tumor location. Additionally, tumor location is an important factor in guiding the use of chemotherapy and radiation therapy 15, and EASC patients had the highest survival rates with preoperative radiation therapy and adjuvant chemotherapy. Preoperative radiation therapy patients had OS rates of 73.8%, 37.0%, and 30.0% at 1, 3, and 5 years, while adjuvant chemotherapy patients had OS rates of 83.3%, 66.7%, and 33.3%. Based on the study results, preoperative radiation therapy combined with adjuvant chemotherapy is recommended for EASC patients, providing important treatment guidance for rare cancers. Furthermore, we constructed a nomogram model to score patients based on our findings, allowing for more accurate treatment decisions for patients.

The strengths of this study include the use of the SEER database to select patients diagnosed with EAM and EASC between 2000 and 2019, providing the first comparative analysis of the incidence and survival prognosis of these two highly studied rare esophageal cancer subtypes. Additionally, we constructed a prognosis model for EAM for the first time, and the AUC value of the constructed EASC prognosis model was superior to that of the model constructed by Qian, Haisheng et al. 19. We also provided treatment recommendations based on a large sample study for the two different rare esophageal cancer subtypes. However, there are limitations to this study. Firstly, it is a retrospective study, which may introduce selection bias. Secondly, specific treatment information was not available in the treatment regimens, making it impossible to provide more specific treatment guidance.

## Conclusion

EAM and EASC are rare esophageal cancer subtypes with limited research. EAM incidence increased until 2014, then decreased; EASC declined until 2017, then rose. Both disproportionately affected males and over 65. These trends underscore the importance of heightened attention and preventive measures specifically targeting EASC. EAM exhibited better overall survival (OS) and cancer-specific survival (CSS) in various AJCC stages, with significant OS differences mainly in stages III-IV. Across pathological grades, EAM patients (grades I-IV) had superior OS and CSS compared to EASC, with significant differences in grades III-IV OS and CSS. EAM patients leaned towards preoperative chemoradiotherapy, while EASC patients were advised preoperative radiation plus full-course chemotherapy. This study enhances our understanding of these rare esophageal cancer subtypes, particularly highlighting the comparatively poorer prognosis associated with EASC subtype compared to EAM and providing valuable treatment insights for clinical decisions.

## Supplementary Material

Supplementary tables.

## Figures and Tables

**Table 1:** Baseline characteristics

	AM	ASC	p
n	377	506	
Age (%)			0.443
< 65	194 (52.5)	246 (48.6)	
≥ 65	183 (48.5)	260 (51.4)	
Sex (%)			0.055
Male	330 (87.5)	418 (82.6)	
Female	47 (13.5)	88 (18.4)	
Race (%)			< 0.001
Asian or Pacific Islander	7 (1.9)	31 (6.1)	
Black	9 (2.4)	33 (6.5)	
Other	5 (1.3)	2 (0.4)	
White	356 (94.4)	440 (87.0)	
Year of diagnosis (%)			<0.001
2000-2004	23 (6.1)	136 (26.9)	
2005-2009	67 (17.8)	125 (24.7)	
2010-2014	153 (40.6)	129 (25.5)	
2015-2019	134 (35.5)	116 (22.9)	
Marital status (%)			0.565
Married	222 (58.9)	287 (56.7)	
Unmarried	155 (41.1)	219 (43.3)	
Tumor size (cm) (%)			0.136
< 2	34 (9.0)	30 (5.9)	
< 4	38 (10.1)	53 (10.5)	
< 6	9 (2.4)	10 (2.0)	
≥ 6	46 (12.2)	44 (8.7)	
Blank(s)	250 (66.3)	369 (72.9)	
Tumor location (%)			< 0.001
Lower third of esophagus	301 (79.8)	305 (60.3)	
Middle third of esophagus	27 (7.2)	107 (21.1)	
Unknown	47 (12.5)	75 (14.8)	
Upper third of esophagus	2 (0.5)	19 (3.8)	
Grade (%)			0.018
I	5 (1.3)	1 (0.2)	
II	25 (6.6)	60 (11.9)	
III	229 (60.7)	303 (59.9)	
IV	5 (1.3)	10 (2.0)	
No/Unknown	113 (30.0)	132 (26.1)	
AJCC Stage (%)			< 0.001
I	13 (3.4)	14 (2.8)	
II	36 (9.5)	18 (3.6)	
III	61 (16.2)	37 (7.3)	
IV	49 (13.0)	64 (12.6)	
Unknown	218 (57.8)	373 (73.7)	
T (%)			<0.001
T1	30 (8.0)	32 (6.3)	
T2	17 (4.5)	13 (2.6)	
T3	66 (17.5)	45 (8.9)	
T4	30 (8.0)	18 (3.6)	
Unknown	234 (62.1)	398 (78.7)	
N (%)			<0.001
N0	59 (15.6)	42 (8.3)	
N1	69 (18.3)	62 (12.3)	
N2	18 (4.8)	14 (2.8)	
N3	13 (3.4)	14 (2.8)	
Unknown	218 (57.8)	374 (73.9)	
M (%)			<0.001
M0	131 (34.7)	85 (16.8)	
M1	49 (13.0)	64 (12.6)	
Unknown	197 (52.3)	357 (70.6)	
Radiation therapy (%)			0.503
Yes	218 (57.8)	280 (55.3)	
No	159	226	
Chemotherapy (%)			0.354
Yes	247 (65.5)	315 (62.3)	
No	130	191	
Surgery (%)			0.02
Yes	124 (32.9)	129 (25.5)	
No	253 (67.1)	377 (74.5)	
Lymph node dissection (%)			<0.001
≤ 3 regional lymph nodes	4 (1.1)	12 (2.4)	
≥ 4 regional lymph nodes	107 (28.4)	80 (15.8)	
No/Unknown	264 (70.0)	414 (81.8)	
nodes removed unknown	2 (0.5)	0 (0.0)	
Metastasis (%)			0.114
Yes	112 (29.7)	177 (35.0)	
No	265 (70.3)	329 (65.0)	
Bone metastasis (%)			0.863
Yes	22 (5.8)	27 (5.3)	
No	355 (94.2)	479 (94.7)	
Liver metastasis (%)			0.085
Yes	26 (6.9)	53 (10.5)	
No	351 (93.1)	453 (89.5)	
Lung (%)			0.056
Yes	14 (3.7)	35 (6.9)	
No	363 (96.3)	471 (93.1)	
Brain metastasis (%)			0.138
Yes	0 (0.0)	5 (1.0)	
No	377 (100.0)	501 (99.0)	
Distant LN metastasis (%)			0.991
Yes	11 (2.9)	16 (3.2)	
No	366 (97.1)	490 (96.8)	
Other metastasis (%)			0.891
Yes	9 (2.4)	14 (2.8)	
No	368 (97.6)	492 (97.2)	

## References

[1] Sung H, Ferlay J, Siegel R L (2021). Global Cancer Statistics 2020: GLOBOCAN Estimates of Incidence and Mortality Worldwide for 36 Cancers in 185 Countries[J]. CA: a cancer journal for clinicians.

[2] Wang S, Zheng R, Arnold M (2022). Global and national trends in the age-specific sex ratio of esophageal cancer and gastric cancer by subtype[J]. International Journal of Cancer.

[3] Lin Y, Wang H-L, Fang K (2022). International trends in esophageal cancer incidence rates by histological subtype (1990-2012) and prediction of the rates to 2030[J]. Esophagus: Official Journal of the Japan Esophageal Society.

[4] Hou H, Meng Z, Zhao X (2019). Survival of Esophageal Cancer in China: A Pooled Analysis on Hospital-Based Studies From 2000 to 2018[J]. Frontiers in Oncology.

[5] Alsop B R, Sharma P (2016). Esophageal Cancer[J]. Gastroenterology Clinics of North America.

[6] Pennathur A, Gibson M K, Jobe B A (2013). Oesophageal carcinoma[J]. Lancet (London, England).

[7] Kushima R (2022). The updated WHO classification of digestive system tumours-gastric adenocarcinoma and dysplasia[J]. Der Pathologe.

[8] Zhang F, Xu B, Peng Y (2023). Incidence and survival of adenocarcinoma with mixed subtypes in patients with colorectal cancer[J]. International Journal of Colorectal Disease.

[9] Fléjou J-F (2011). [WHO Classification of digestive tumors: the fourth edition][J]. Annales De Pathologie.

[10] Zhao X, Li Y, Yang Z (2021). Adenocarcinoma with Mixed Subtypes in the Early and Advanced Gastric Cancer[J]. Canadian Journal of Gastroenterology & Hepatology.

[11] Sheng H, Wei X, Mao M (2019). Adenocarcinoma with mixed subtypes is a rare but aggressive histologic subtype in colorectal cancer[J]. BMC cancer.

[12] Han J P, Hong S J, Kim H K (2015). Long-term outcomes of early gastric cancer diagnosed as mixed adenocarcinoma after endoscopic submucosal dissection[J]. Journal of Gastroenterology and Hepatology.

[13] Evans M, Liu Y, Chen C (2017). Adenosquamous Carcinoma of the Esophagus: An NCDB-Based Investigation on Comparative Features and Overall Survival in a Rare Tumor[J]. Oncology.

[14] Zhang P, Dong S, Sun W (2023). Deciphering Treg cell roles in esophageal squamous cell carcinoma: a comprehensive prognostic and immunotherapeutic analysis[J]. Frontiers in Molecular Biosciences.

[15] Cheng C, Luo Z, Xiong W (2022). Epidemiology and survival outcomes in adenosquamous carcinoma: a population-based study[J]. International Journal of Colorectal Disease.

[16] Gamboa A C, Meyer B I, Switchenko J M (2020). Should adenosquamous esophageal cancer be treated like adenocarcinoma or squamous cell carcinoma?[J]. Journal of Surgical Oncology.

[17] Ota H, Yokoyama S, Kawai K (2021). [A Resected Case of Adeno-Squamous Carcinoma of Gallbladder with Liver Invasions]. Gan to Kagaku Ryoho.

[18] Quirynen R, Ocak S, Duplaquet F (2023). Long-term complete remission after severe pembrolizumab-induced immune-related encephalitis in metastatic lung adeno-squamous carcinoma: A case report[J]. Respiratory Medicine Case Reports.

[19] Qian H, Ji X, Liu C (2021). Clinical Characteristics, Prognosis, and Nomogram for Esophageal Cancer Based on Adenosquamous Carcinoma: A SEER Database Analysis[J]. Frontiers in Oncology.

[20] Ni P-Z, Yang Y-S, Hu W-P (2016). Primary adenosquamous carcinoma of the esophagus: an analysis of 39 cases[J]. Journal of Thoracic Disease.

[21] Zhang H D, Chen C G, Gao Y Y (2014). Primary esophageal adenosquamous carcinoma: a retrospective analysis of 24 cases[J]. Diseases of the Esophagus: Official Journal of the International Society for Diseases of the Esophagus.

[22] Doll K M, Rademaker A, Sosa J A (2018). Practical Guide to Surgical Data Sets: Surveillance, Epidemiology, and End Results (SEER) Database[J]. JAMA surgery.

[23] Huang Z, Wang J, Zhang R (2023). Pancreatic adenosquamous carcinoma: A population level analysis of epidemiological trends and prognosis[J]. Cancer Medicine.

[24] van der Kaaij R T, Koemans W J, van Putten M (2020). A population-based study on intestinal and diffuse type adenocarcinoma of the oesophagus and stomach in the Netherlands between 1989 and 2015[J]. European Journal of Cancer (Oxford, England: 1990).

[25] Schizas D, Kapsampelis P, Mylonas K S (2018). Adenosquamous Carcinoma of the Esophagus: A Literature Review[J]. Journal of Translational Internal Medicine.

[26] Nagtegaal I D, Odze R D, Klimstra D (2020). The 2019 WHO classification of tumours of the digestive system[J]. Histopathology.

[27] Ahadi M, Sokolova A, Brown I (2021). The 2019 World Health Organization Classification of appendiceal, colorectal and anal canal tumours: an update and critical assessment[J]. Pathology.

[28] Korphaisarn K, Morris V, Davis J S (2019). Signet ring cell colorectal cancer: genomic insights into a rare subpopulation of colorectal adenocarcinoma[J]. British Journal of Cancer.

[29] Zhu Y, Thandar M, Cheng J (2023). Comparison of survival outcomes and survival prediction in patients with primary colorectal MANEC and primary colorectal SRCC: a population-based propensity-score matching study[J]. Journal of Cancer Research and Clinical Oncology.

[30] Sijben J, Peters Y, Bas S (2023). Dutch individuals' views of screening for oesophageal cancer: a focus group study[J]. BMJ open gastroenterology.

[31] Chen S-B, Weng H-R, Wang G (2013). Primary adenosquamous carcinoma of the esophagus[J]. World Journal of Gastroenterology.

[32] Chen S-B, Liu D-T, Chen Y-P (2022). Surgical resection for esophageal adenosquamous carcinoma: an analysis of 56 cases[J]. World Journal of Surgical Oncology.

[33] Rice T W, Lerut T E M R, Orringer M B (2016). Worldwide Esophageal Cancer Collaboration: neoadjuvant pathologic staging data[J]. Diseases of the Esophagus: Official Journal of the International Society for Diseases of the Esophagus.

