# Supplementary material for: Occurrence and Prognosis of Mixed Subtype Adenocarcinoma and Adeno-Squamous Carcinoma in Esophageal Cancer
Source: J Cancer. 2024 Jan 20;15(5):1442–61. doi: 10.7150/jca.92230 (PMC10861812; doi:10.7150/jca.92230)
Supplement: Supplementary file 1 — Supplementary tables. [file jcav15p1442s1.pdf]

ST1: AM nomogram score

	Points
Chemotherapy	
Yes	41
No/Unknown	66
Surgery	
Surgery	9
no surgery	66
Lymph node dissection	
>=4 regional lymph nodes	66
No/Unknown	71
<=3 regional lymph nodes	74
nodes removed unknown	100
Other metastases	
No/Unknown	66
Yes	81
Distant LN metastases	
No/Unknown	66
Yes	74
Liver metastases	
No/Unknown	66
Yes	99
Bone metastases	
No/Unknown	66
Yes	79
AJCC Stage	
I-II	66
StageUnknown	84
III-IV	96
Tumorlocation	
Upper third of esophagus	0
Unknown	65
Lower third of esophagus	66
Middle third of esophagus	90
Marital status	

Married	66
Unmarried	76
Race	
Black	22
White	43
Asian or Pacific Islander	66
Other	71
Sex	
Male	66
Female	82
Age	
<65	66
>=65	76

Total Points	Pr(Survival months< 12)	Pr(Survival months<36)	Pr(Survival months< 60)
740	0.0372	0.0986	0.138
760	0.0594	0.1545	0.2133
780	0.0942	0.2376	0.3215
800	0.1478	0.3549	0.4657
820	0.2277	0.5077	0.6369
840	0.3414	0.6819	0.8056
860	0.4909	0.8429	0.9291
880	0.6642	0.9498	0.9861
900	0.8286	0.9921	0.999
920	0.9422	0.9996	1
940	0.99	1	1
960	0.9994	1	1

ST2：ASC nomogram score

	Points
Chemotherapy	
Yes	15
No/Unknown	46
Radiation therapy	
Yes	31
No/Unknown	46
Surgery	
Surgery	0
no surgery	46
nodes removed unknown	63

Brain metastases	
No/Unknown	46
Yes	100
Lung metastases	
No/Unknown	46
Yes	66
Liver metastases	
No/Unknown	46
Yes	51
Bone metastases	
No/Unknown	46
Yes	77
Grade	
I-II	46
No/Unknown	53
III-IV	56
Stage	
I-II	46
StageUnknown	63
III-IV	67
Tumorlocation	
Upper third of esophagus	34
Middle third of esophagus	43
Lower third of esophagus	46
Unknown	49
Marital status	
Married	46
Unmarried	57
Sex	
Male	45
Female	46
Age	
<65	46
>=65	50

Total Points	Pr(Survival months< 12)	Pr(Survival months<36)	Pr(Survival months< 60)
500	0.1458	0.3019	0.3543
520	0.2166	0.427	0.4923
540	0.315	0.5781	0.6502
560	0.4436	0.7375	0.8037

580	0.5969	0.8741	0.9198
600	0.7553	0.9597	0.9799
620	0.8871	0.9931	0.9977
640	0.966	0.9996	0.9999
660	0.9947	1	1
680	0.9997	1	1
700	1	1	1
720	1	1	1
